# Prussian Blue Tablets for Cesium Decorporation: pH-Dependent Performance Under Normogastric and Hypochlorhydric Conditions in an In Vitro Gastrointestinal Model

**DOI:** 10.3390/ph18111702

**Published:** 2025-11-10

**Authors:** Borja Martínez-Alonso, Guillermo Torrado Durán, Hugo Pardo Laurel, Víctor Guarnizo-Herrero, M. Ángeles Peña Fernández

**Affiliations:** Department of Biomedical Sciences, Faculty of Pharmacy, University of Alcalá (UAH), Campus Universitario, Crta. Madrid—Barcelona km. 33.600, 28771 Alcalá de Henares, Spain; borja.martineza@uah.es (B.M.-A.); guillermo.torrado@uah.es (G.T.D.); hugo.pardo@edu.uah.es (H.P.L.); victor.guarnizo@uah.es (V.G.-H.)

**Keywords:** Prussian blue, in vitro gastrointestinal model, tablets, antidote, target, cesium decorporation

## Abstract

**Background/Objectives**: Prussian blue (PB) is the agent of choice for internal cesium (Cs) decorporation, yet its performance is critically dependent on gastrointestinal (GI) pH and formulation attributes. Hypochlorhydria, common in patients treated with proton pump inhibitors, may alter the stagewise distribution of Cs binding during GI transit. This study aimed to compare the performance of different PB formulations in sequential in vitro models simulating normogastria and hypochlorhydria: normogastric regimen (NG) and hypochlorhydric regimen (HC). **Methods**: A static, enzyme-free sequential model was applied using compendial simulated fluids (SGFs pH 1.2 or acetate pH 4.0, SIF pH 6.8, and phosphate buffer pH 7.2). The formulations tested included PB active pharmaceutical ingredient (API) (bulk), compression blend, PB tablets 500 mg (PB tablets), and Radiogardase^®^. For each stage, cesium bound (qs, mg/g PB), fractional contributions (fs), and total capture (qtotal) were quantified. Additional analyses included sensitivity to initial Cs concentration (C_0_) and desorption in mineralized water. **Results**: Overall performance was primarily determined by formulation (*p* < 0.0001), with a significant formulation × regimen interaction. The compression blend and PB tablets exhibited the highest decorporation capacity, PB-API showed intermediate performance, and Radiogardase^®^ was clearly lower. Under HC, capture was concentrated in the gastric stage (44–47%), whereas in NG, it shifted toward intestinal stages. Desorption in the mineralized water was statistically significant but negligible compared with total capture, supporting the stability of cesium sequestration. **Conclusions**: Formulation and gastric acidity regimens not only determine the total cesium capture but also redistribute it across the GI tract. PB tablets represent an effective and accessible alternative to Radiogardase^®^, maintaining robust decorporation capacity under clinically relevant pH conditions.

## 1. Introduction

Radiological incidents and internal exposures to radio nuclides have underscored the need for effective countermeasures to reduce the body burden of ^137^Cs and ^134^Cs. The Goiânia accident (1987) and other historical events demonstrated both the environmental persistence of radioactive cesium and the value to accelerate their elimination [1,2,3]. In clinical practice and official guidance, Prussian blue (PB; insoluble Prussian Blue, ferric hexacyanoferrate) is the decorporation agent of choice for internal contamination with cesium (and thallium), supported by regulatory approval and operational use in real-world scenarios [2,3,4,5]. The reference medicinal product, Radiogardase^®^, marketed with restricted global availability, is supplied as 500 mg hard capsules. Its product information and regulatory labeling specify indications, dosing, and precautions, with multi-week administration schedules depending on internal burden and clinical monitoring [2,6,7,8]. Together with response manuals (CDC/REMM, UKHSA/HPA, IAEA), these sources consolidate the role of PB within preparedness plans and strategic stockpiles for radiological emergencies [3,4,9,10].

The mechanism of action of PB is linked to its hexacyanoferrate framework: a cubic network (Fe^3+^-CN-Fe^2+^) with zeolitic properties and hydrated interstitial sites that can exchange monovalent cations (Cs^+^, Tl^+^) for H^+^/K^+^ and trap them in superficial interstitial cavities of the lattice [10,11]. When administered orally, PB is not significantly absorbed; it acts intraluminally and interrupts the enterohepatic recirculation of cesium excreted in bile, thereby promoting fecal elimination [2,3,4]. At the population level and in clinical case series (including the Goiânia accident), its use is associated with marked reductions in the biological half-life of ^137^Cs, accelerating internal decontamination [1,5,12]. In practice, guidelines recommend starting treatment as early as possible (ideally within the first hours), maintaining it for at least 30 days, and performing biological and analytical monitoring to adjust the duration [2,3,8]. The safety profile is favorable (constipation being the most common adverse effect), and logistical and clinical precautions are clearly defined in official product information and regulatory resources [2,13,14]. Consistent with this labeling, Radiogardase^®^ is recommended to be taken with meals (fed state), as food stimulates bile secretion and enterohepatic circulation and may facilitate cesium excretion; however, neither the SmPC nor US labeling (DailyMed) provides regimen-specific instructions related to gastric acidity (e.g., normogastria vs. hypochlorhydria), and a focused literature search likewise did not identify such guidance [13,14].

A key factor in the performance of PB is its dependence on pH and on certain critical quality attributes. Pharmaceutic and regulatory studies have shown that the binding capacity of PB for Cs^+^ is minimal under strongly acidic conditions (pH 1–2 and maximal near pH 7–7.5 with additional sensitivity to contact time, particle size, and solid-state water content [7,8,10,15]. Ionic competition (Rb^+^, K^+^, Na^+^, Ca^2+^, Mg^2+^) and the composition of the surrounding matrix (salinity/hardness) also influence capture efficiency and can even promote desorption in some settings, as described in model systems using PB hydrogels, aerogels, and composites [16,17,18,19,20,21]. These findings suggest that the pH history and prior exposure to cations during gastrointestinal (GI) transit may modulate the stagewise distribution of cesium capture and, consequently, the overall decorporation capacity.

From a GI physiology standpoint, segmental pH profiles and their fasted/postprandial variability are well-characterized: fasted gastric pH typically lies around 1.2–2.0 whereas duodenal pH is 6–6.8, with appreciable temporal and interindividual fluctuations [22,23,24]. These conditions are altered in subpopulations with hypochlorhydria (e.g., due to chronic proton pump inhibitor (PPI) use, atrophic/autoimmune gastritis, age), leading to sustained elevations of gastric pH toward values at or above 4.0 [25,26,27]. At the population level, the prevalence of Helicobacter pylori, which can contribute to altered acidity, remains high in many regions, with geographic gradients and temporal declines that add heterogeneity to baseline gastric acidity [28,29]. Moreover, extensive PPI use across Europe (with issues of under- and over-utilization) and demographic trends (aging) suggest that hypochlorhydria is a clinically relevant scenario rather than a marginal condition [25,26,30,31,32]. Accordingly, contrasting normogastria and hypochlorhydria warrants attention when evaluating PB performance in vitro.

To design reproducible models, various scientific and regulatory sources provide standardized parameters for in vitro (static) digestion or dissolution tests that specify pH, medium composition, and phase times (gastric and intestinal), and these are widely applied in pharmaceutical development [33,34,35,36,37]. Such frameworks enhance comparability across studies; however, by fixing conditions stage by stage, they do not themselves capture the impact of the loading history, what occurs in one stage influences the next, nor the sequential competition for binding sites. Consequently, there is methodological interest in decomposing Cs capture into sequential stages with incremental pH values that mimic the GI tract and in analyze the interaction between formulation and gastric acidity regimen, determining both total and stagewise amounts of Cs bound/removed [38,39].

In the recent literature, numerous studies have explored PB analogs and advanced systems for Cs capture in environmental or biomedical contexts: nanostructured PB, PB–alginate composites, porous hydrogels and aerogels, or Prussian blue analogs (PBAs, e.g., CuFe) with complementary properties including reactive oxygen species (ROS) scavenging [16,17,18,19,20,21,40,41]. These contributions document sensitivity to medium chemistry, ionic competition, and pH trends, and point toward the optimization of adsorbent materials. Nevertheless, they do not systematically and comparatively address, within a pharmaceutical framework, how the dosage form (e.g., tablets versus capsules) and the sequential GI pH history, where capture is redistributed across stages, account for differences in total decorporation capacity. Nor do they typically isolate the effect of prior saturation under intestinal pH conditions, or desorption under mineralized water (a clinically relevant condition for recommendations on fluid intake during treatment). This translational gap, between materials science and real oral pharmaceutical use under GI conditions, provides the rationale for the present study [11,16,17,18,19,20,21,33,34,35,36,37,38,39,40,41,42].

In parallel with advances in materials science, steps have been taken to develop and characterize new pharmaceutical forms of PB with industrial feasibility and broader accessibility. Recently, the physicochemical and pharmacotechnical characterization of PB for tablet formulation has been reported, focusing on critical quality parameters (particle size, water content, crystalline phase, compressibility) and on the development, characterization, and stability of 500 mg PB tablets as an open access alternative to Radiogardase^®^ [15,43]. In the regulatory and clinical practice arenas, the Summary of Product Characteristics (SmPC) and drug database records (BfArM/Heyl, DailyMed) remain key references for indications, dosing, and precautions, while IAEA and WHO preparedness and emergency-response resources reinforce the role of PB as an essential component of radiological countermeasures [13,14,44,45]. The toxicology of cesium and its biological distribution further support the rationale for using PB to interrupt intestinal reabsorption and shorten systemic retention [12].

Based on the foregoing, we designed an in vitro study using a static, sequential model to evaluate how gastric acidity regimen and formulation influence the decorporation capacity for Cs, understanding by regimen the sequence of three pH stages applied in the in model: a gastric stage (simulated gastric fluid (SGF) pH 1.2 to represent normogastria or acetate buffer pH 4.0 to represent hypochlorhydria) followed by two intestinal stages (simulated intestinal fluid (SIF) pH 6.8 and phosphate buffer pH 7.2). Therefore, two different regimens are compared: normogastric regimen (NG), pH 1.2 → 6.8 → 7.2 versus hypochlorhydric regimen (HC), pH 4.0 → 6.8 → 7.2. NG and HC differ solely in the gastric pH. The analysis integrates a global metric (q_total_) with its stagewise decomposition (q_s_) and fractions (f_s_), assesses sensitivity to the initial Cs concentration in the medium (C_0_), incorporates single-stage controls to probe prior saturation, and characterizes desorption in mineralized water. Stage-wise retention is reported as the percentage (*w*/*w*) of the total Cs dose retained (%R_s_) by the correspondent sample at the end of the stage s, for both the sensitivity analysis and the single-stage controls. This approach, grounded in different methodological frameworks and in evidence of PB’s pH-dependence and ionic competition, connects capture mechanisms with formulation parameters and plausible GI scenarios, providing a pharmaceutical comparative framework that is currently lacking in the literature [7,8,9,11,16,17,18,19,20,21,33,34,35,36,37,38,39,40,41,42]. Without presupposing outcomes, and based on the literature, it is anticipated that a less acidic gastric pH (4.0) will increase early-stage (S1) capture relative to pH 1.2, which could in turn affect the residual capacity for S2–S3 given PB’s finite ion-exchange capacity and documented saturation/site-occupancy effects; under some conditions, higher early capture at pH 4.0 may reduce subsequent capture and even the total. The present work tests this expectation under a pre-specified experimental design and statistical analysis [7,22,23,24,30,31,32,33,34,35,36,37,38,39,46].

## 2. Results and Discussion

### 2.1. General Overview

This section provides an overview of the experimental design and statistical analysis strategy. We first report the primary endpoint, the overall decorporation capacity q_total_ (mg Cs/g PB), followed by a stage-wise decomposition of capture q_s_ and the corresponding contribution fractions f_s_ = q_s_/q_total_. We then assess the sensitivity to the initial concentration C_0_ using a three-factor GLM on %R_s_ and evaluate single-stage controls to isolate the prior-loading effects observed in the sequential regimen. Finally, we quantify desorption in mineralized water and document inter-stage Milli-Q washes. This structure progresses from the overall effect (total capacity) to a mechanistic breakdown (by stage), and then to supporting experiments that probe loading history, concentration dependence, and reversibility.

Assumptions and robustness checks: across ANOVAs/GLM (all with n = 3 per cell), residual Q-Q plots were acceptable, and Levene’s tests were generally non-significant; where variance heterogeneity was detected (single-stage pH 6.8), the group separation was large and the qualitative conclusions (Single ≈ NG-S2 > HC-S2) did not depend on the homoscedasticity assumption. Multiple comparisons were controlled with Tukey HSD (reported as letters), and effect directions were coherent across endpoints (q_total_, q_s_, f_s_, %R_s_). Each Results subsection specifies the corresponding test/post hoc procedure. As negative controls, placebo samples showed ≤1% decorporation, i.e., at or below the assay’s uncertainty floor; these values are treated as negligible and are not used in capture calculations, confirming minimal background and the absence of spurious “carry-over”.

### 2.2. Overall Decorporation Capacity (q_total_)

#### 2.2.1. General Overview (Two-Way ANOVA)

**Figure 1:**
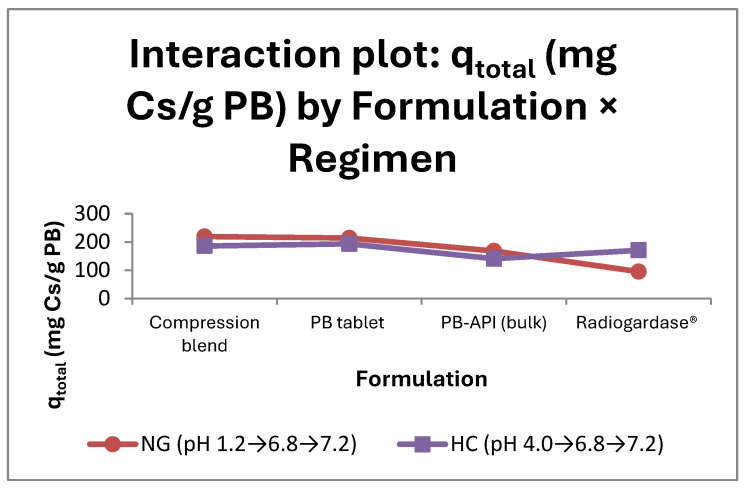
Overall decorporation capacity (q_total_, mg Cs/g PB) by formulation under two GI regimens. Interaction plot (formulation × regimen) showing LS means with 95% intervals based on the residual MSE from the two-way ANOVA (GML, Type III SS) (df = 16; n = 3 per cell; α = 0.05). Regimens: NG (pH 1.2 → 6.8 → 7.2) and HC (pH 4.0 → 6.8 → 7.2). In NG, compression blend and PB tablets achieve higher q_total_ than PB-API (bulk) and Radiogardase^®^; under HC, Radiogardase^®^ increases but remains below compressed formulations, consistent with a significant interaction. Note: Placebo controls (no PB) are not shown because q_total_ is normalized per gram of PB; placebo removal was ≤1% which falls within the method’s analytical uncertainty/error floor; therefore, they were excluded from calculations. For compression blend and PB tablets, the NG–HC shift appears modest; nevertheless, simple-effects tests within the regimen indicate significant NG > HC differences (α = 0.05; see Table 1). Although HC may seem to contradict the “less acid facilitates capture” statement, that refers to S1; the total depends on stage-wise capacity/saturation (see Section 2.3 and Section 2.5).

The GLM with factors formulation (four levels) and regimen (NG and HC) explained virtually all variability in the primary endpoint (R^2^ = 97.44%). There was a highly significant main effect of formulation and a highly significant formulation × regimen interaction, whereas the main effect of regimen was not significant (Type III: formulation F(3,16) = 132.05, *p* < 0.0001; regimen F(1,16) = 0.24, *p* = 0.629; interaction F(3,16) = 70.50, *p* < 0.0001), indicating that the NG-HC shift does not change the overall mean when formulation is not specified, yet it does alter the relative ranking across formulations (i.e., an interaction) (Table S1). At an equal PB dose (75 mg per vessel), the overall performance is therefore driven primarily by formulation [15,43], and the effect of regimen is formulation dependent. Non-parallel lines in the interaction plot support this interpretation; accordingly, we proceeded with simple-effects analyses (Figure 1).

**Table 1:** q_total_ (mg Cs/g PB): mean ± SD (pooled) and 95% CI by formulation within each regimen.

Formulation	NG—Mean (mg Cs/g PB)	NG—95% CI (mg Cs/g PB)	HC—Mean (mg Cs/g PB)	HC—95% CI (mg Cs/g PB)	Δ (NG-HC) (mg Cs/g PB)
Compression blend	219.07	211.46–226.69	185.69	173.71–197.68	+33.38
PB tablets	214.01	206.39–221.63	193.15	181.16–205.13	+20.86
PB-API (bulk)	168.16	160.54–175.77	140.68	128.70–152.66	+27.48
Radiogardase^®^	95.04	87.42–102.66	170.69	158.70–182.67	−75.65

Notes. 95% CIs are from one-way ANOVAs within each regimen (pooled SD per regimen; n = 3 per group). The Δ(NG-HC) column is computed directly from the reported means.

Statistics for this section: two-way ANOVA (GLM, Type III sums of squares), Lesser Squares (LSs) means ± 95% CI; α = 0.05; n = 3 per cell; assumptions checked as in Section 2.1.

#### 2.2.2. Simple Effects One-Way ANOVA

By regimen (Formulation within each regimen; Tukey):

- NG (pH 1.2 → 6.8 → 7.2): clear differences (one-way ANOVA F(3,8) = 302.6; *p* < 0.0001). Means (mg/g): Compression blend 219.07, PB tablets 214.01, PB-API 168.16, Radiogardase^®^ 95.04. Tukey: Compression blend ≈ PB tablets, and both > PB-API > Radiogardase^®^ (all other pairwise comparisons significant) (Table S2). Assumptions acceptable (Levene *p* = 0 0.299).
- HC (pH 4.0 → 6.8 → 7.2): differences detected (one-way ANOVA F(3,8) = 19.95; *p* = 0.0005). Means (mg/g): PB tablets 193.15, Compression blend 185.69, Radiogardase^®^ 170.69, PB-API 140.68. Tukey: PB-API < (Radiogardase^®^, Compression blend, PB tablets); Radiogardase^®^ vs. Compression/PB tablets and Compression vs. PB tablets were not significant (Table S3). Assumptions acceptable (Levene *p* = 0 0.082).

Matrix of means (NG vs. HC) and NG-HC contrasts (see Table 1):

- Compression blend: NG 219.07 vs. HC 185.69 → Δ = +33.38 mg/g; *p* = 0.0007 (NG > HC)
- PB tablets: NG 214.01 vs. HC 193.15 → Δ = +20.86 mg/g; *p* = 0.011 (NG > HC)
- PB-API (bulk): NG 168.16 vs. HC 140.68 → Δ = +27.47 mg/g; *p* = 0.0017 (NG > HC)
- Radiogardase^®^: NG 95.04 vs. HC 170.69 → Δ = −75.65 mg/g; *p* = 0.0018 (HC > NG)

In NG, Compression blend and PB tablets clearly outperform PB-API and—by a wide margin—Radiogardase^®^. In HC, PB tablets/compression remain above PB-API, while Radiogardase^®^ exhibits an intermediate level (not different from Tablet/Blend). These patterns are consistent with the strong Formulation × Regimen interaction in the two-way model (Tables S2 and S3).

#### 2.2.3. Integrated Interpretation

1. Formulation dominated overall performance: at an equal PB dose, PB tablets and Compression blend consistently outperform PB-API; Radiogardase^®^ is far lower in NG and moderate in HC [15,43].
2. Regimen is formulation-dependent: there is no global NG vs. HC shift, but the ordering and effect sizes change by formulation (strong interaction).
3. Practical implication: under acidic gastric conditions (NG), PB tablets and Compression blend show robust performance, whereas Radiogardase^®^ exhibits lower total capacity. Under less acidic conditions (HC), Radiogardase^®^ improves and approaches the PB tablets and Compression blend (without surpassing them in NG) [10,15].

Under the present in vitro conditions and equalized PB dose, a greater body clearance of Cs is expected with PB tablets than with Radiogardase^®^ in a normogastric scenario. In hypochlorhydria, PB tablets and Radiogardase^®^ show broadly similar performance. These conclusions are bound by the in vitro model and warrant confirmation considering the complexity of in vivo GI transit.

The similarity between PB tablets and Compression blend indicates that the compression step itself does not impair PB’s decorporation capacity. The advantage of the both of them over PB-API is their plausible compatibility with changes in particle size/specific surface induced during preparation (e.g., sieving), which could increase accessible exchange sites; this mechanism should be framed as a plausible hypothesis and supported with appropriate references [10,15,47,48].

Additionally, excipient effects may contribute: the microcrystalline cellulose in the tablet matrix can facilitate the rapid disintegration and dispersion of PB particles upon wetting, increasing the effective surface available for ion exchange, an effect repeatedly documented for microcrystalline cellulose-based tablets and other systems [49,50].

### 2.3. Stage-Wise Analysis

**Figure 2:**
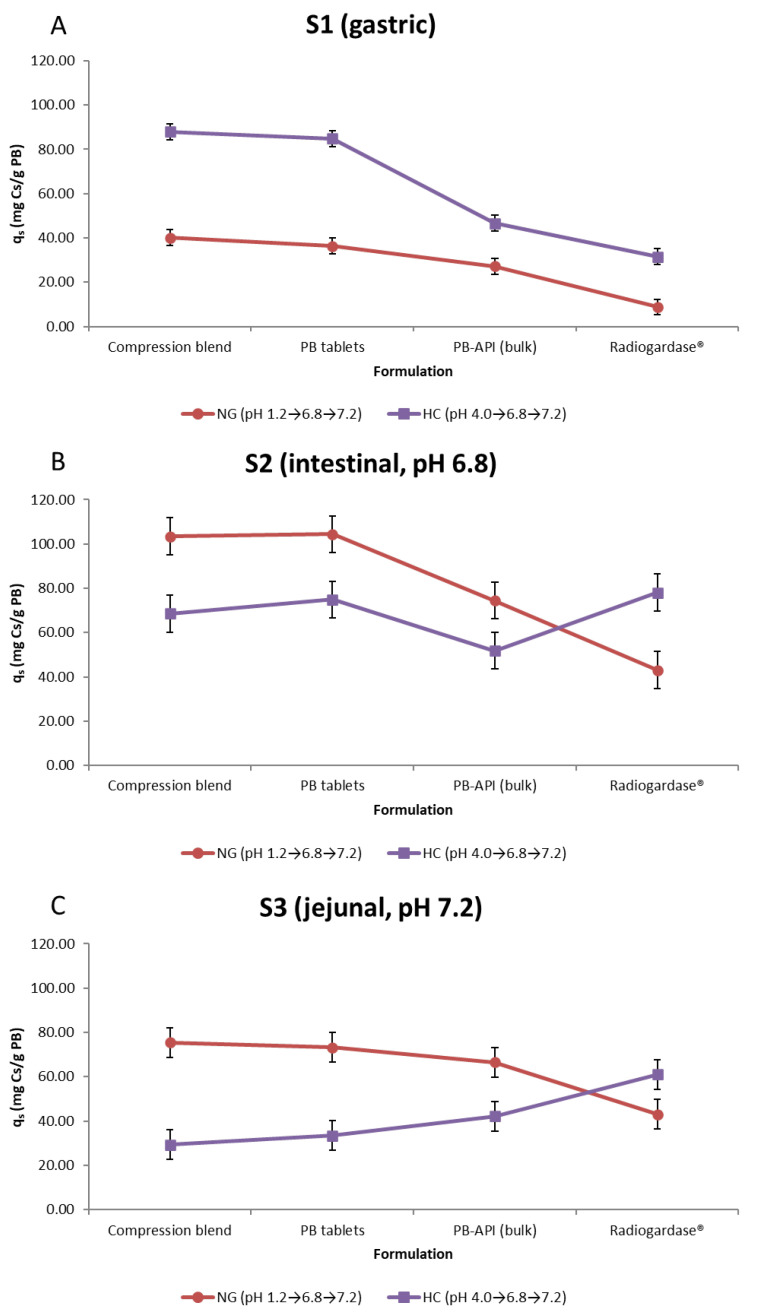
Stage-wise capture (q_s_, mg Cs/g PB) by formulation under NG and HC. (**A**) S1 (gastric); (**B**) S2 (intestinal, pH 6.8); (**C**) S3 (jejunal, pH 7.2). Points are LS means from two-way ANOVA (Formulation × Regimen) fitted separately for each stage; error bars are 95% intervals based on the residual MSE (df = 16; n = 3 per cell). Regimens: NG (pH 1.2 → 6.8 → 7.2) and HC (pH 4.0 → 6.8 → 7.2). Overall, uptake concentrates in S1 under HC and shifts to S2–S3 under NG, with Compression blend and PB tablets ranking highest in NG; Radiogardase^®^ increases notably in later stages under HC. These patterns explain the interaction observed for q_total_. (Tables S5–S7). Clarification: Although S2 (pH 6.8) and S3 (pH 7.2) are identical across regimens, NG–HC differences at these stages reflect the regimen-dependent carryover from S1 (site occupancy/saturation) rather than pH per se; see Section 2.3 and Section 2.5 for details. Formulation note: Although S2 (pH 6.8) and S3 (pH 7.2) are identical across regimens, Radiogardase^®^ shows a distinct HC ≥ NG profile across stages compared with the other formulation. This likely reflects formulation/API attributes that influence dispersion and the accessible surface (e.g., particle-size distribution, solid-state water, and the absence of a disintegrant matrix), together with a more modest S1 increase under HC than in compressed formulations, limiting pre-saturation and preserving S2–S3 (see Section 2.3 and Section 2.5).

To delineate how the total capacity (q_total_) is distributed, and how uptake at each stage influences subsequent stages under NG or HC, we analyzed stage-wise capture (q_S1_ gastric, q_S2_ intestinal pH 6.8, q_S3_ jejunal pH 7.2) using a two-way ANOVA (Formulation × Regimen), and summarized LS means with 95% CI, fitted separately for each stage (Figure 2A–C). Statistics for this section: two-way ANOVA per stage (GLM, Type III SS), LS means ± 95% CI; df = 16; n = 3 per cell; α = 0.05; assumptions checked as in Section 2.1.

#### 2.3.1. Stage-Wise Results

S1 (gastric). The model exhibited a marked Formulation × Regimen interaction (Type III: Regimen F(1,16) = 852.36, *p* < 0.0001; Formulation F(3,16) = 304.67, *p* < 0.0001; Interaction F(3,16) = 43.87, *p* < 0.0001). Averaging across regimens, the Compression blend and PB tablets showed the highest means, followed by PB-API, with Radiogardase^®^ clearly lower. Least squares (LS) means (mg/g): Compression blend 64.01, PB tablets 60.60, PB-API 36.92, Radiogardase^®^ 20.20. The pattern is consistent with HC > NG (62.74 vs. 28.13 mg/g) in S1 and substantial formulation differences are already at the gastric compartment (Table S8A,B). Tukey grouped Compression ≈ PB tablets > PB-API > Radiogardase^®^.

S2 (pH 6.8). The main effects of Regimen and Formulation, and their interaction, were significant (Regimen F(1,16) = 21.78, *p* = 0.0003; Formulation F(3,16) = 29.25, *p* < 0.0001; Interaction F(3,16) = 33.76, *p* < 0.0001). Overall, NG > HC for q_S2_ (81.34 vs. 68.33 mg/g). PB tablets and Compression blend formed the high-uptake group; PB-API and Radiogardase^®^ the lower group (Table S9A,B) Compression vs. PB tablets was not significant by Tukey.

S3 (pH 7.2). A strong Regimen effect (NG > HC; LS means ≈ 64.60 vs. 41.49 mg/g) and a significant interaction were observed; unlike S1 and S2, the main effect of Formulation was not significant (Type III: Regimen F(1,16) = 108.07, *p* < 0.0001; Formulation F(3,16) = 0.21, *p* = 0.885; Interaction F(3,16) = 42.20, *p* < 0.0001), and Tukey’s test did not separate formulations when regimens were averaged (Table S10A,B).

Considering the stage-wise ANOVAs, S1 uptake is consistently higher under HC than NG across all formulations (Figure 2A), in line with reduced capture at strongly acidic pH 1.2 versus milder acidity at pH 4.0. At S2 (pH 6.8), q_s_ is higher under NG for Compression blend and PB tablets, consistent with more available sites after the lower S1 of NG; Radiogardase^®^, however, departs from this pattern and shows HC ≥ NG (Figure 2B). By S3 (pH 7.2), the cumulative pH history dominates: NG > HC for compressed formulations and PB-API, whereas Radiogardase^®^ remains higher under HC (Figure 2C). This stage-wise shift explains the interaction observed in q_total_: for PB tablets and Compression blend, the lower S1 in NG is compensated, often exceeded, by gains at S2–S3; Radiogardase^®^ follows an opposite regimen pattern and therefore its total increases under HC. The NG-HC decomposition by stage makes this explicit (Figure 2A–C).

#### 2.3.2. NG-HC Decomposition by Stage (Δq_s_)

For each formulation, the difference Δq_s_ = q_s_(NG) − q_s_(HC) shows how the total Δ is built:

- Compression blend: Δq_S1_ − 47.80, Δq_S2_ + 34.94, Δq_S3_ + 46.24 = Δq_total_ + 33.38.
- PB tablets: Δq_S1_ − 48.52, Δq_S2_ + 29.49, Δq_S3_ + 39.88 = Δq_total_ + 20.86.
- PB-API (bulk): Δq_S1_ − 19.41, Δq_S2_ + 22.60, Δq_S3_ + 24.27 = Δq_total_ + 27.47.
- Radiogardase^®^: Δq_S1_ − 22.72, Δq_S2_ − 34.99, Δq_S3_ − 17.94 = Δq_total_ − 75.65.

For Compression blend, PB tablets and PB-API, NG reduces S1 but increases S2–S3 enough to compensate (or exceed) the total. For Radiogardase^®^, all stages are lower under NG and the total decreases (Table 2).

#### 2.3.3. Contribution Fractions (f_S_)

**Figure 3:**
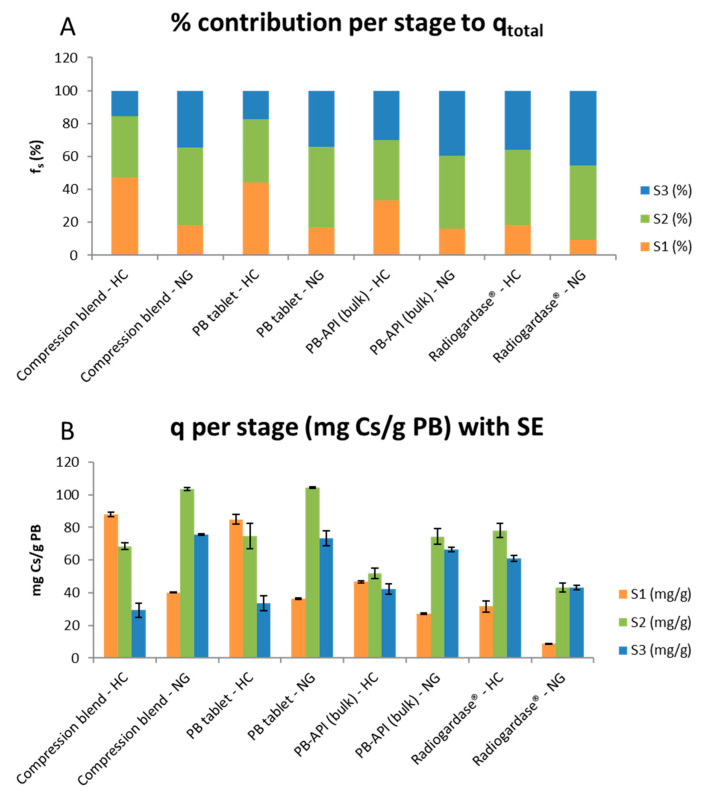
Stagewise distribution of cesium capture by formulation and gastric-acidity regimen. (**A**) Fractional contribution (% f_s_) of each stage (S1, S2, S3) to the total cesium captured (q_total_) NG and HC conditions. Bars are stacked by stage; values represent mean percentages (n = 3). (**B**) Absolute cesium captured (q_s_, mg Cs/g PB) at each stage (mean ± 95% CI, n = 3) for the same regimens and formulations. The comparison shows that, under HC, the Compression blend and PB tablets concentrate a larger share in S1 (44–47%), whereas under NG, these formulations and PB-API shift capture towards S2–S3 (S2 ≈ 44–49%, S3 ≈ 34–40%). Radiogardase^®^ also shifts toward S2–S3 in NG but with lower absolute q_s_, so it does not maintain the same q_total_.

The share of the total contributed by each stage highlights how the distribution shifts with prior exposure and regimen (means per combination) (Figure 3A):

- Compression blend: HC f_S1_ 47.34%, f_S2_ 36.89%, f_S3_ 15.76%; NG f_S1_ 18.31%, f_S2_ 47.22%, f_S3_ 34.47%.
- PB tablets: HC 43.94%/38.76%/17.30%; NG 16.98%/48.76%/34.25%.
- PB-API: HC 33.14%/36.86%/30.00%; NG 16.19%/44.28%/39.54%.
- Radiogardase^®^: HC 18.49%/45.75%/35.76%; NG 9.30%/45.35%/45.35%.

#### 2.3.4. General Pattern and Discussion

Under HC, Pb tablets and Compression blend concentrate a large share in S1 (44–47%). Under NG, PB tablets, Compression blend and PB-API shift to S2–S3 (S2 44–49%; S3 34–40%). Radiogardase^®^ also shifts to S2–S3 in NG (45%/45%), but with lower absolute q_s_, so it does not sustain q_total_. (Table 3: f_S1_, f_S2_, f_S3_ with SE/95% CI; Figure 3A,B: stacked bars by combination).

- HC favors early capture (S1) for Tablet, Compression blend, and PB-API due to the higher initial pH in S1, which then occupies PB sites heading into S2 and S3.
- NG favors later capture (S2–S3) because little is removed in S1. This raises the total for Tablet, Compression blend, and PB-API by preserving most capture sites for the stages closer to the optimal pH range.
- Radiogardase^®^ loses across all stages under NG, with the largest penalty in S2 (−35 mg/g). The % captured in S1 differs less between regimens, leaving S2 similar in % terms and providing no compensation in S3; consequently, q_total_ declines. From a pharmaceutical standpoint, the compressed formulations (PB tablets and Compression blend) incorporate a microcrystalline-cellulose matrix that promotes rapid disintegration/dispersion and increases the effective surface of PB; preparation steps (e.g., sieving, compression) may also narrow the particle-size distribution. Radiogardase^®^ was tested using the capsule content at equal PB mass, but its API is not synthesized at UAH (unlike the PB used for the developed formulations); therefore, intrinsic API differences may contribute to its distinct regimen-dependent profile (HC ≥ NG) and to lower absolute q_s_ at later stages, despite identical S2/S3 pH and the normalization per gram of PB.

This stage-wise and fractional analysis supports the primary endpoint: formulation determines performance, and regimen re-allocates the dominant stage of capture.

### 2.4. C_0_ Sensitivity Analysis

#### 2.4.1. Results (GLM C_0_ × Stage × Regimen on %R_s_)

**Figure 4:**
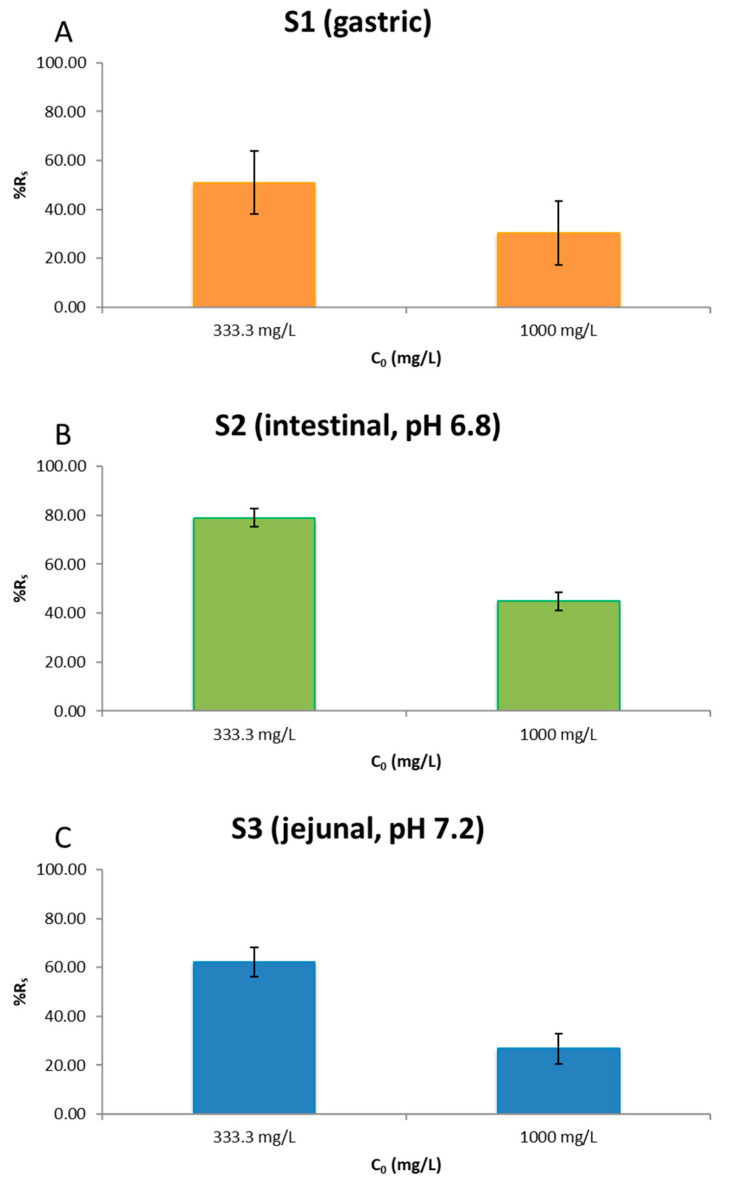
Stage-wise LS means ± 95% CI of %R_s_ for C_0_ = 1000 and 333.3 mg/L: (**A**) S1 (gastric), (**B**) S2 (intestinal, pH 6.8), (**C**) S3 (jejunal, pH 7.2). Estimates from the GLM (C_0_ × Stage × Regimen) with residual df = 24. The *Y* axis is common (0–100%) across panels to ease comparison.

A three-factor GLM showed C_0_ and Stage as highly significant main effects, whereas Regimen was not significant as a main effect (Type III: C_0_ F = 600.95, *p* < 0.0001; Stage F = 113.80, *p* < 0.0001; Regimen F = 1.85, *p* = 0.186). Significant interactions were detected for C_0_ × Stage (*p* = 0.0001), Stage × Regimen (*p* < 0.0001) and the three-way C_0_ × Stage × Regimen (*p* = 0.0001). Model fit was excellent (R^2^ = 98.23%, SE = 3.69; residual df = 24) (Table S11). At the global level (LS means), lowering C_0_ from 1000 to 333.3 mg/L increased %R_s_ by approximately 30 percentage points (33.93% → 64.05%). By stage (averaged over regimens), LS means shifted as follows: S1 30.30% → 51.02%, S2 44.81% → 79.03%, S3 26.68% → 62.10% when moving from 1000 to 333.3 mg/L, indicating pronounced sensitivity to C_0_, particularly in later stages (Figure 4). Three-factor GLM (Type III SS), LS means ± 95% CI; α = 0.05; n = 3 per cell; Residual normality (Q–Q) and homoscedasticity (Levene) were checked as specified in the Methods; no variance heterogeneity was detected. Because C_0_ has two levels, the pairwise comparison reduces to the mean difference with 95% CI (equivalent to the stage-level ANOVA contrast); no Welch correction was required.

#### 2.4.2. One-Way ANOVA by Stage (Factor: C_0_)

- S1 (gastric): means 51.02% (333.3 mg/L) vs. 30.30% (1000), one-way ANOVA F = 3.35, *p* = 0.097 (trend). Tukey not significant at 5% (Δ = 20.72 ± 25.21).
- S2 (pH 6.8): means 79.03% (333.3) vs. 44.81% (1000), one-way ANOVA significant, *p* < 0.001. Tukey significant (Δ = 34.22 ± 11.27).
- S3 (pH 7.2): means 62.10% (333.3) vs. 26.68% (1000), one-way ANOVA significant, *p* = 0.002. Tukey significant (Δ = 35.42 ± 18.97).

Unlike S2 and S3, the S1 increase did not reach significance: despite S1 being less favorable for Cs capture at lower pH, the magnitude of the improvement upon lowering C_0_ was insufficient to meet the 5% threshold (Table S12).

#### 2.4.3. Discussion

Reducing C_0_ increased the overall %R_s_ by ~30 percentage points, a robust GLM result consistent across regimens (non-significant C_0_ × Regimen). This implies that with a lower initial Cs load, PB utilizes its capacity more effectively along the sequence.

The significant C_0_ × Stage interaction and the stage-wise ANOVAs show that most of the gain from lowering C_0_ occurs in S2 and S3. This aligns with the stage-wise analysis in mg/g: when S1 is constrained by acidity, S2/S3 can sustain, or even raise, the total provided because those later stages are not already capacity-limited by earlier capture.

### 2.5. Single-Stage Controls

#### 2.5.1. pH 6.8 Test: Single vs. S2 of NG and HC

A one-way ANOVA on %R_s_ (three groups: Single, NG-S2, HC-S2) showed significant differences (F = 16.65; *p* = 0.0036; n = 3 per group). Means (±SE, LSD): HC-S2 37.43% ± 2.31; NG-S2 52.18% ± 2.31; Single 55.02% ± 2.31. Tukey: Single ≈ NG-S2 (not significant), and both > HC-S2 (*p* < 0.05). Levene was significant (*p* = 0.026), indicating unequal variances; nonetheless, the contrast of HC-S2 vs. Single/NG-S2 was large and consistent. Thus, at pH 6.8, the Single sample does not outperform NG-S2 (no appreciable prior saturation in NG because S1 at pH 1.2 removes little), but it clearly exceeds HC-S2 (in HC, S1 at pH 4 removes more and “occupies” capacity before S2) (Table S13A–C).

#### 2.5.2. pH 7.2 Test: Single vs. S3 of NG and HC

A one-way ANOVA (groups: Single, NG-S3, HC-S3) was highly significant (F = 184.6; *p* < 0.0001). Means (±SE): HC-S3 16.71% ± 1.92; NG-S3 36.65% ± 1.92; Single 68.48% ± 1.92. Tukey: all three pairs differ (Single > NG-S3 > HC-S3; *p* < 0.05). Levene was not significant (*p* = 0.181). These results indicate that, at pH 7.2, the Single sample exceeds both sequence stages, clearly evidencing prior saturation in the sequential pathway (the sample reaches S3 with part of PB capacity already bound) (Table S14A–C).

#### 2.5.3. Single 6.8 vs. Single 7.2

A one-way ANOVA with factor pH (6.8 vs. 7.2) was significant (F = 145.2; *p* = 0.0003). Means: Single 6.8: 55.02% ± 0.79; Single 7.2: 68.48% ± 0.79. Tukey: difference 13.47% (*p* < 0.05).

When Cs captured by PB is assessed without prior exposure to earlier stages, pH 7.2 yields a higher percentage removal than pH 6.8 with a fresh sample, consistent with PB’s greater affinity/operational capacity for Cs^+^ in neutral to slightly basic media.

As observed, both single-stage tests show higher decorporation than their regimen-matched counterparts within NG or HC. The only exception is Single 6.8 vs. NG-S2 (pH 6.8), where no advantage is seen for the single stage because the NG sequence incurs minimal prior saturation (S1 at pH 1.2 removes little Cs) (Table S15A–C).

**Figure 5:**
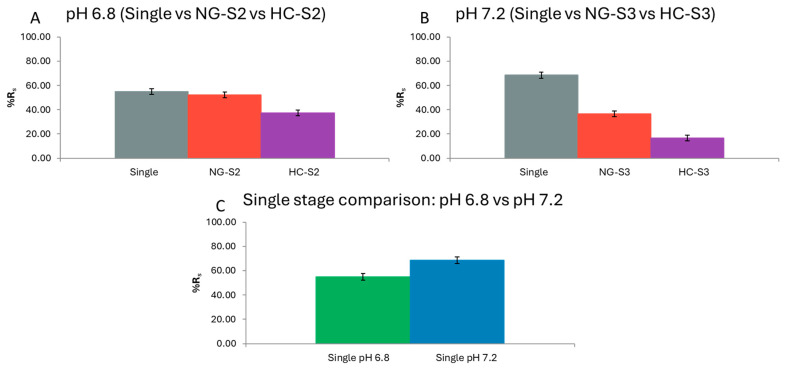
Single-stage controls of %R_s_. (**A**) pH 6.8: Single, NG-S2, HC-S2 (one-way ANOVA F = 16.65, *p* = 0.0036; Tukey: Single ≈ NG-S2 > HC-S2). (**B**) pH 7.2: Single, NG-S3, HC-S3 (F = 184.6, *p* < 0.0001; Tukey: Single > NG-S3 > HC-S3). (**C**) Single pH: 6.8 vs. 7.2 (F = 145.2, *p* = 0.0003; Tukey: Δ = 13.47 percentage points). Bars show means ± 95% CI; n = 3 per group. (Common *Y* axis range 0–100% for comparability).

Moreover, while earlier sections showed greater Cs capture at pH 6.8 than pH 7.2 within the sequential assays, the single-stage comparison reverses this: capture is significantly higher at pH 7.2 than at pH 6.8, aligning with published reports on the pH-dependent Cs capture of PB (Figure 5).

Viewed together with the C_0_ sensitivity analysis, these findings indicate that PB’s Cs-binding capacity is saturable: the more Cs a PB sample has bound in earlier stages, the less it will capture in subsequent ones, even under otherwise favorable conditions.

These controls reinforce the stage-wise and two-way q_total_ conclusions: the tract location where the largest capture occurs depends on exposure history. If transit begins with a capacity-consuming step (e.g., HC-S1 at pH 4), later stages (S2 and S3) perform worse. By contrast, a fresh sample at pH 7.2 exhibits higher effective capacity. This explains why NG shifts capture toward S2–S3 and, for formulations such as PB tablets and Compression blend, can maintain or increase total capture, whereas Radiogardase^®^ fails to compensate at later stages.

### 2.6. Desorption in Mineralized Water

#### 2.6.1. Desorption Results

**Figure 6:**
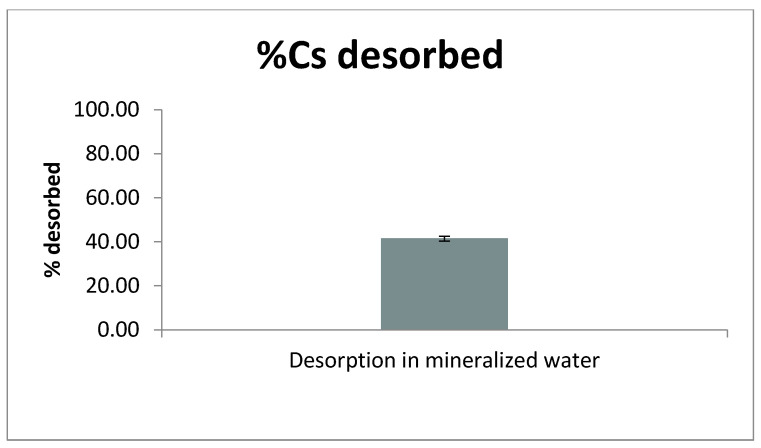
Desorption in mineralized water. Bar shows the mean percent desorbed (41.37%) with 95% CI 40.27–42.48 (n = 3). One-sided non-inferiority vs. 0% was met (lower 95% bound 40.62%; *p* < 0.0001). Inter-stage Milli-Q washes were <LOD. (For completeness, the corresponding desorbed amount had a mean 88.55 mg Cs/g PB, lower 95% bound 82.615 mg/g; *p* = 0.0003.).

Desorbed percentage: Analysis of one sample set (n = 3) yielded a mean desorption of 41.37% (SD 0.44; 95% CI 40.27–42.48%). The range was narrow (40.97–41.85%), indicating good repeatability across replicates (Figure 6).

Desorbed amount (mg Cs/g PB). Expressed as mass, the mean desorbed amount was 88.55 mg Cs/g PB (SD 3.52), with a 95% CI lower bound of 82.62 mg/g.

Non-inferiority vs. zero. For both % desorbed and mg Cs/g PB, one-sample non-inferiority tests against 0%/0 mg/g were clearly significant (*p* < 0.0001 and *p* = 0.0003, respectively), with 95% CI lower bounds above zero. Thus, desorption different from zero is demonstrated under the mineralized water condition tested.

Comparator in non-mineralized water (inter-stage washes). In all inter-stage washes with Milli-Q water, the Cs released was “not detected” (<LOD), with no substitutions applied. These washes are not used for capture calculations and serve solely to document the absence of measurable carry-over/desorption in very low mineralized water.

#### 2.6.2. Discussion

A mean desorption of ~41% (≈ 89 mg/g) from PB preloaded with Cs when contacted with a multication solution representative of high mineralized water provides clear evidence that Na^+^/K^+^/Ca^2+^/Mg^2+^ can competitively displace Cs^+^ from the PB lattice under realistic ionic strength conditions. The narrow 95% CI and the noninferiority tests against zero confirm that the effect is not marginal but quantitatively relevant.

These findings have clinical implications: because mineralized water promotes Cs release, the administration of PB should avoid hard/high mineralized waters. Very soft/low mineralized water should be preferred to minimize ionic competition during and immediately after GI transit.

They also carry environmental/safety implications: the substantial desorption observed indicates that the uncontrolled disposal of PB-containing residues into mineralized receiving waters is inappropriate, as it could remobilize Cs. Dedicated containment and treatment consistent with radiological and local regulations are warranted.

Finally, the “not detected” outcomes in all inter-stage Milli-Q washes support this interpretation: in non-mineralized water, we observed no measurable desorption, reinforcing both the clinical recommendation and the waste-management caution above.

### 2.7. Real-World Considerations and Future Work Perspectives

Within the constraints of this in vitro model, our findings support practical, label-aligned advice: administer PB with meals and avoid hard/high-mineralized waters, preferring very soft/low-mineralized water to minimize ionic competition during and shortly after GI transit. The stage-wise results also suggest that gastric acidity can redistribute capture across S1–S3, which may help interpret variability in patients with hypochlorhydria. These points are intended to inform discussion; prospective clinical studies would be required before considering any labeling changes.

For future work and formulation perspectives, a desirable goal is a PB dosage form with reduced regimen dependence (more consistent performance under NG and HC) to improve usability and patient compliance. Potential strategies include staged/dual release (combining an immediate-release fraction with a delayed/enteric fraction) to temper early S1 uptake under HC and preserve S2–S3; multilayer or multiparticulate systems to modulate wetting/dispersion; microenvironmental pH modifiers/buffering layers to stabilize the PB microenvironment; and particle-engineering to tune accessible surface and diffusion without provoking early over-sorption. In light of the observed C_0_-sensitivity, dose fractionation (smaller, more frequent doses) also merits evaluation as a use pattern. These avenues are hypothesis-generating and warrant dedicated pharmaceutical and clinical studies.

## 3. Materials and Methods

### 3.1. Materials and Reagents

Prussian blue (batch DFO-2009-083) was supplied by the Center for Applied Chemistry and Biotechnology, University of Alcalá (UAH, Spain). Excipients included glyceryl behenate (Compritol^®^ 888 ATO, CAS 91052-55-0, Gattefossé, Madrid, Spain), hydroxypropyl methylcellulose (HPMC, CAS 9004-65-3, Guinama, Valencia, Spain), polyvinylpyrrolidone K-30 (PVP K-30, CAS 9003-39-8, Thermo Fisher Scientific, Madrid, Spain), and microcrystalline cellulose (CAS 9004-34-6, Thermo Fisher Scientific, Madrid, Spain).

Radiogardase^®^ 500 mg hard capsules (batch 24002125; Heyl Chemisch-pharmazeutische Fabrik GmbH & Co. KG, Berlin, Germany) were purchased for comparison.

Analytical grade reagents were used: Milli-Q water was produced with a Milli-Q purification system (Millipore, Billerica, MA, USA), hydrochloric acid 37%, extra pure (batch 12069508, Scharlau, Barcelona, Spain), potassium phosphate monobasic ≥99% (KH_2_PO_4_, batch A0441958, Thermo Fisher Scientific, Madrid, Spain), potassium phosphate dibasic anhydrous ≥99% (K_2_HPO_4_, batch A0450732, Thermo Fisher Scientific, Madrid, Spain), phosphoric acid ACS reagent, ≥85% in H_2_O (batch MKCT0237, Sigma-Aldrich, Madrid, Spain), cesium chloride ≥99% (CsCl, batch Z05F018, Thermo Scientific, Madrid, Spain), cesium single-element standard solution, 1000 mg/L (as CsNO_3_ in H_2_O) for ICP (ref. CE01080100, Scharlau, Barcelona, Spain), glacial acetic acid, reagent grade ACS/ISO (batch 95442, Scharlau, Barcelona, Spain), sodium acetate trihydrate (batch 141632, Panreac AppliChem, Barcelona, Spain), sodium chloride USP/ACS grade (batch 0000252176, Thermo Fisher Scientific, Madrid, Spain), potassium chloride ACS grade (batch 243980, Thermo Fisher Scientific, Madrid, Spain), calcium chloride dihydrate ACS reagent (batch A0408607, Thermo Fisher Scientific, Madrid, Spain), and magnesium chloride hexahydrate ACS grade (batch 220636, Thermo Fisher Scientific, Madrid, Spain). All solutions were prepared with Milli-Q water.

Falcon™ 50 mL conical tubes were purchased from Sigma-Aldrich (Madrid, Spain). Volumetric flasks, vials, beakers, pipettes, and graduated cylinders, all manufactured from borosilicate glass, were obtained from Scharlab (Madrid, Spain).

### 3.2. Formulations Under Test

Five sample types were evaluated under the experimental conditions detailed in the section (Sequential GI capture assay).

The developed PB tablets were evaluated alongside Radiogardase^®^ as the reference medicinal product. Bulk PB (API) was included to isolate the intrinsic sorption performance of the API, independent of excipients, and to compare it with the formulation. The compression blend (tablet blend before compaction) was assayed together with the finished tablet to examine whether the compression step per se modifies PB decorporation capacity. A placebo blend (tablet excipients without PB) served as a negative control to verify that any Cs removal is attributable to PB rather than to excipients.

PB tablets correspond to a direct-compression, immediate-release, uncoated formulation containing 71.43% (*w*/*w*) PB with microcrystalline cellulose, povidone K-30, hydroxypropyl methylcellulose, and glyceryl behenate (see Table 4). This blend was qualified for direct compression by SeDeM analysis (adequate acceptance indices; good flow/compressibility: angle of repose 32.11°, flow time < 1 s), and manufactured as 500 mg PB tablets with good pharmacotechnical properties, rapid disintegration (<1 min), a validated assay, and content uniformity and confirmed stability per ICH conditions. By contrast, Radiogardase^®^ is a hard gelatin capsule whose fill is PB (no disintegrant matrix). Immediate-release tablets provide early fragmentation into a high-surface-area particulate cloud aided by the excipient matrix, whereas capsule products release a bulk powder fill after shell opening/dissolution, which could lead to slower humectation [8,9,15,43].

To enable a fair comparison across dosage forms, the mass of PB active pharmaceutical ingredient (API) (m_PB_) per test vessel was equalized to a target value of 75 mg. For each formulation, the mass of product weighed in each test was therefore adjusted according to its PB mass fraction (w_PB_) according to the following equation:

$$m_{product,test}=\frac{m_{PB}}{w_{PB}}$$

We set m_PB_ = 75 mg because this corresponds to weighing approximately 105 mg of the tablet when the mean tablet mass is 700 mg and contains 500 mg PB (w_PB_ = 500/700 = 0.714). Crucially, this choice preserves the PB/solution ratio used in the standard decorporation capacity characterization of PB tablets and in stability testing [43]: one whole 700 mg tablet (500 mg PB) in 100 mL of CsCl 1000 mg/L gives 5 mg PB/mL, our assay uses 75 mg PB in 15 mL, resulting in the same 5 mg PB/mL. This ensures the direct comparability of capacities across formulations under GI-sequential conditions (Table 4).

1. API (PB-API). Bulk insoluble PB powder (batch DFO-2009-083), used as received without excipients.
2. PB tablets (batch LPAP20221DE3). Manufactured at the Pharmaceutical Technology research laboratories (UAH). The complete manufacturing process and characterization have been reported in [43].
3. Radiogardase^®^ 500 mg hard capsules (batch 24002125). Commercial reference product containing 500 mg of insoluble PB per capsule (manufacturer’s specification), plus approved pharmaceutical excipients.
4. Compression blend. Homogeneous dry blend of PB with mannitol, HPMC, PVP K-30, and microcrystalline cellulose.
5. Placebo. Excipient blend matching the tablet excipient composition without PB (negative control). By definition, w_PB_ = 0, placebo served as control and to analyze possible retention of Cs^+^ on the excipients.

### 3.3. Preparation of Test Samples

All sample types were handled to deliver the same mass of active PB per test vessel (m_PB_ = 75 mg), adjusting each product mass by its w_PB_, specified in Table 5. Unless otherwise stated, samples were used as received and manipulated with minimal mechanical stress to avoid artifacts.

- PB-API (bulk powder). No further preparation was required. The bulk PB powder was weighed directly to provide 75 mg of PB to each vessel.
- PB tablets. The tablets used belonged to one of the batches that were manufactured by direct compression as described in detail by Martínez-Alonso et al. [43]. The formula of these tablets is specified in Table 4. For the GI assays, the test portions of approximately 105 mg of tablet (which corresponds to 75 mg of PB) were obtained by mechanically subdividing an intact PB tablet and weighing individual fragments on a calibrated analytical balance (Mettler AM10, Mettler, Spain). Portions for each test where chosen and weighted together so they weight approximately 105 mg; in all cases, the exact set of fragments mass was recorded and used to compute PB content and to normalize outcomes, so results did not depend on hitting 105 mg exactly. The tablet disintegrated in < 1 min, ensuring that fragmentation did not limit PB exposure and wetting during the tests; therefore, the measured decorporation capacity reflects the intrinsic PB performance rather than tablet disintegration. Prior assay/content-uniformity data [43] support the representativeness of the set of fragments weighted.
- Radiogardase^®^ 500 mg hard capsules. Capsules were opened and the capsule contents were weighed to deliver m_PB,test_ (75 mg of PB). This decision was made to improve the comparability of the different samples. The study aim is decorporating capacity of PB, not disintegration performance. The gelatin shell does not contribute to ion exchange and dissolves rapidly in gastric media, using the fill only ensures that the effects depend only on PB behavior and keeps the experiment comparable to tablet fragments and blends, where equal PB mass is tested.
- Compression blend. A single laboratory scale bat of 1 kg of compression blend matching the tablet excipient ratios (Table 1) was prepared following the same order used before tableting, full method can be found in Martínez Alonso et al. [43]. Briefly, all components were weighed (Kern KB 360-3N, Kern & Sohn, Balingen, Germany). All ingredients, except the lubricant/glidant (glyceryl behenate), were blended for 5 min at 40 rpm in a PP Sabadell OX (type 41411) V-blender (P. Prat S.A., Sabadell, Spain). Glyceryl behenate was then added and blending continued for 2 min at 60 rpm to avoid over-lubrication. The mass weighed for each assay was calculated from w_PB_ (Table 5) to deliver m_PB,test_.
- Placebo. Prepared identically to the compression blend without PB, using the excipient ratios shown in Table 4. Placebo served as a negative control and matrix blank, and no dose equalization was applied (w_PB_ = 0).

### 3.4. Simulated GI Media

All media were enzyme-free and were prepared with freshly produced Milli-Q water. Media were equilibrated at 37 ± 0.5 °C, and their pH was verified at 37 °C using a calibrated pH meter GLP 22 (Crison Instruments s.a., Barcelona, Spain) with buffers pH 1.00, 4.01 and 7.00. To isolate pH-driven effects on Prussian blue, sodium chloride was not added to gastric media.

- Gastric medium (normogastric), pH 1.20 ± 0.05 (SGF, enzyme-free).
- The medium consisted of a solution of hydrochloric acid with sodium chloride (pH 1.2, osmolality ≈ 180 mOsm/kg), prepared by acidifying purified water with concentrated HCl (37%) to pH 1.20 ± 0.05 at 37 °C.
- Gastric medium (hypochlorhydria), pH 4.00 ± 0.05.
- The medium consisted of a 50 mM acetate buffer prepared from sodium acetate trihydrate and adjusted with glacial acetic acid to pH 4.00 ± 0.05 at 37 °C.
- Intestinal medium, pH 6.80 ± 0.05 (SIF, enzyme-free).
- The medium consisted of a 50 mM phosphate buffer prepared from KH_2_PO_4_/K_2_HPO_4_ and adjusted to pH 6.80 ± 0.05 at 37 °C.
- Jejunal medium, pH 7.20 ± 0.05.
- The medium consisted of a 50 mM phosphate buffer prepared from KH_2_PO_4_/K_2_HPO_4_ and adjusted to pH 7.20 ± 0.05 at 37 °C.

All media were freshly prepared on the day of use and contained 1000 mg/L CsCl. Details of their sequential application (stage duration, volumes and transfers) are described in the following section.

### 3.5. Sequential GI Capture Assay

Two sequential regimens were implemented to represent the transit of Prussian blue (PB) formulations through the human GI tract. In the NG, samples were exposed consecutively to pH 1.2, 6.8 and 7.2, representing gastric, intestinal and neutral/jejunal environments. Because PB’s decorporation capacity is strongly pH-dependent, a second regimen was evaluated to reflect patients receiving antacids or proton-pump inhibitors (PPIs), which develop a hypochlorhydria state. Therefore, the HC was established using pH 4.0, 6.8 and 7.2. Both sequences employed fresh medium at each stage under controlled temperature and agitation to enable a fair, stage-by-stage comparison.

**Figure 7:**
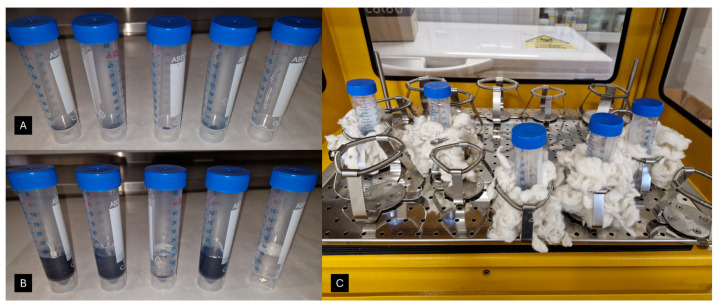
Photographic overview of sample handling in the in vitro assays. (**A**) Labeled 50 mL Falcon^®^ tubes containing the weighed test portions before medium addition. Samples from left to right: PB API, Compression blend, PB tablets, Radiogardase^®^, and placebo. (**B**) Same tubes immediately after adding the test medium; the PB-containing solid fraction is visible at the bottom. (**C**) Tubes secured on the orbital shaker during incubation (37 °C, 100 rpm). Images illustrate the setup used in the sequential GI-regimen assays.

**Figure 8:**
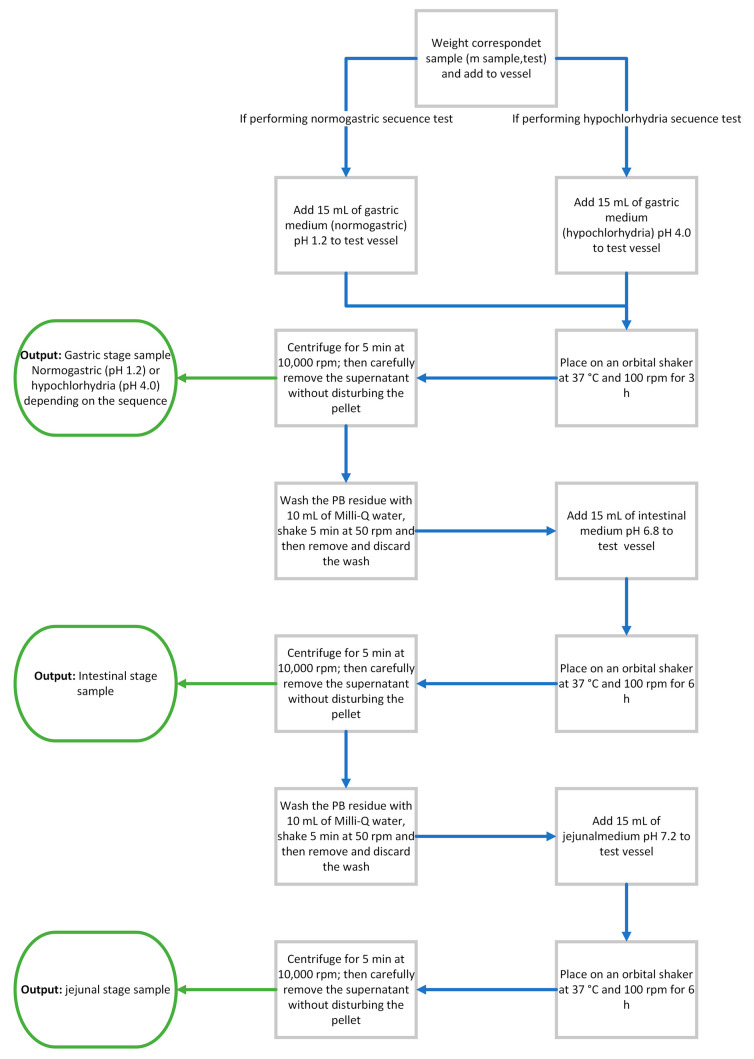
Process flow of the sequential GI capture assay (NG and HC). Blue arrows indicate a process to be followed. Green arrows indicate an output obtained.

For each experiment, the corresponding m_product,test_ for each formula was placed in labeled vessels (50 mL polypropylene conical centrifuge tubes, Falcon^®^; blank controls confirmed no measurable Cs^+^ adsorption to the tube walls) and processed at 37 °C under orbital shaking. A photographic overview of representative samples at key steps, prior to medium addition, immediately after addition, and during orbital agitation, is provided Figure 7. For clarification, a process flow for both regimens is included (Figure 8). The following procedure was performed:

- Gastric stage (S1) (NG: pH 1.2 or HC: pH 4.0). Fifteen microliters of the appropriate gastric medium (pH = 1.2 in NG and pH = 4 in HC) was added to the vessel. Samples were then shaken on an orbital shaker (Thermoshake, Gerhardt, Königswinter, Germany) at 100 rpm and 37 °C for 3 h. Subsequently, the suspension was centrifuged (Jouan b 3.11 centrifuge, Saint-Herblain, France) for 5 min at 10,000 rpm, and the supernatant was carefully collected (gastric stage sample) without disturbing the centrifugation sediment (sedimented solid fraction of PB-containing solids, with excipients where applicable, obtained after centrifugation). The PB residue was washed with 10 mL of Milli-Q water, shaken for 5 min at 50 rpm, and the wash was removed and discarded.
- Intestinal stage (S2) (pH 6.8, 6 h). After the wash, 15 mL of intestinal medium (pH 6.8) were added to the same vessel. Samples were shaken at 100 rpm and 37 °C for 6 h, centrifuged for 5 min at 10,000 rpm, and the supernatant was collected (intestinal stage sample). The centrifugation sediment was washed with 10 mL of Milli-Q water, shaken for 5 min at 50 rpm, and the wash was discarded.
- Jejunal stage (S3) (pH 7.2, 6 h). The PB residue was then contacted with 15 mL of pH 7.2 medium, shaken at 100 rpm and 37 °C for 6 h, centrifuged for 5 min at 10,000 rpm, and the supernatant was collected (jejunal stage sample).

In addition to the sequential regimens, two single-stage controls were performed using fresh PB tablet fragments (105 mg per test, providing 75 mg PB). One control was exposed only to pH 6.8 and the other only to pH 7.2. Each test used 15 mL of the corresponding medium containing CsCl (1000 mg/L), was shaken at 100 rpm and 37 °C for 6 h, and was then centrifuged (5 min, 10,000 rpm) and processed identically to the sequential stages. These controls were designed to assess whether PB exhibits progressive saturation across stages and whether the decorporating capacity at a given pH is equal to or higher when a fresh tablet sample is tested in a single stage, compared with the corresponding stage within the sequential protocol.

For both regimens, an additional sensitivity experiment was performed using PB tablets (test portions 105 mg, providing 75 mg PB). The CsCl working solution (1000 mg/L) used in the sequential assay was diluted 1:3 (*v*/*v*) with Milli-Q water to obtain 333.3 mg/L. All other conditions (stage volumes, temperature, shaking speed, and stage durations) were identical to the sequential protocol. Supernatants were processed and analyzed as described under sampling and sample handling. This experiment was designed to assess how the initial cesium concentration (C_0_) influences capture in each stage and in total, and to examine potential PB saturation during sequential exposure.

### 3.6. Sampling and Sample Handling

At the end of each stage, as well as for the blank media controls, the single-stage controls (pH 6.8 only; pH 7.2 only), and the sensitivity runs (C_0_ = 333.3 mg/L), the collected supernatants were processed identically. A 5.0 mL aliquot was withdrawn and filtered through a 0.45 µm nylon membrane into clean vials. Then, 2.5 mL of the filtrate was diluted to 25.0 mL with Milli-Q water in class-A volumetric flasks (1:10 dilution). The resulting solutions were submitted for cesium analysis by ICP-OES for cesium determination.

### 3.7. Competitive Ion/Desorption Test in Mineralized Waters

After completing the GI sequence, a desorption assay was conducted to evaluate whether common cations in mineralized waters could displace previously captured cesium. For the PB tablet experiments, three independent tests (n = 3) were performed. In each test, approximately 100 mg of tablet residue (the solid recovered from a single 105 mg tablet portion after sequential GI exposure) was transferred to a clean vessel and contacted with 15 mL of a multication solution formulated to represent high mineralization drinking water, using intermediate concentrations within realistic upper ranges [51,52]: Na 75 mg/L, K 3.5 mg/L, Ca 110 mg/L, and Mg 35 mg/L. The solution was prepared from analytical grade salts as follows (per liter of water): NaCl 190.7 mg, KCl 6.68 mg, CaCl_2_·2H_2_O 403.4 mg, and MgCl_2_·6H_2_O 292.8 mg. Suspensions were maintained on an orbital shaker at 37 °C and 100 rpm for 60 min, after which the supernatants were collected and submitted to ICP-OES for cesium determination. Desorbed Cs was expressed as both the total amount and the percentage of Cs released related to the total previously captured Cs by that same sample in the sequential assay.

To provide a comparator under very low ionic strength, inter-stage wash waters (10 mL Milli-Q per transfer) were collected at each stage, filtered (0.45 µm nylon), diluted 1:10 with Milli-Q, and analyzed by ICP-OES (Cs 697.3 nm) under the same instrumental conditions as the test supernatants. Concentrations below LOD were recorded as “<LOD” without substitution, as wash fractions were not used in capture calculations; their purpose was solely to verify the absence of desorption/carry-over in non-mineralized water.

### 3.8. ICP-OES Analytical Method

Cesium was quantified by ICP-OES using a Varian 720-ES instrument (Varian, Inc., Palo Alto, CA, USA) equipped with an autosampler. Operating conditions were as follows:

- Power: 1.00 kW.
- Coolant flow 15.0 L/min.
- Auxiliary gas flow 1.5 L/min.
- Nebulizer gas flow 1.0 L/min.
- Integration time 10 s.
- Emission line for Cs 697.3 nm.
- The rest of the equipment parameters were operated with factory-default optics purge/temperature and RF oscillator settings; no non-standard hardware or user adjustments were applied.

An external multi-point calibration was prepared from the certified 1000 mg/L Cs standard by serial dilution in Milli-Q water to cover the expected sample range, including a 0 mg/L blank. A linear regression with a free intercept was fitted and its goodness-of-fit was verified before sample analysis.

Alongside the samples, reagent blanks and a mid-level QC solution at 50 mg/L Cs (prepared from the same stock standard) were analyzed. The QC was run at the beginning and end of each analytical batch and periodically during the sequence to verify continuing calibration; recoveries were tracked and remained within acceptance criteria for routine ICP-OES practice.

No further dilution beyond the 1:10 preparation was required; all measurements fell within the calibrated range.

The LOD and LOQ were established from 10 independent blank injections. Using the blank standard deviation and the calibration slope (LOD = 3·σ/S; LOQ = 10·σ/S), the method LOD and LOQ were 0.4 mg/L and 0.9 mg/L, respectively. These values correspond to 4.0 mg/L (LOD) and 9.0 mg/L (LOQ) in the original solutions before the 1:10 dilution.

### 3.9. Endpoints and Calculations

#### 3.9.1. Primary Endpoint

For each test, the total amount of Cs captured across the sequential GI assay was computed as

$$q_{total}=\frac{\sum_{s=1}^{3}\left(C_{0,s}-C_{s}\right)V_{s}}{m_{PB}}$$

where C_0_,_s_ and C_s_ correspond, respectively, to the initial and final Cs concentrations (mg/L) in each stage of the regimen (S1, S2, S3), V_s_ is the volume of the stage (L), and mPB is the mass of active PB used in the regimen (0.075 g). The corresponding mass of Cs decorporated/captured (mg) per experiment is given as follows:

$$m_{captured}=q_{total}\cdot m_{PB}$$

#### 3.9.2. Stage-Wise Analysis

Stage-wise capacity and removal were calculated as q_s_ and %R_s_ as follows:

$$q_{s}=\frac{\left(C_{0,s}-C_{s}\right)V_{s}}{m_{PB}}$$

$${\%R}_{s}=100(1-\frac{C_{s}}{C_{0,s}})$$

Contribution fractions, i.e., which fraction from q_total_ belongs to each stage, were

$$f_{s}=\frac{q_{s}}{q_{total}}$$

with f_1_ + f_2_ + f_3_ = 1.

The regimen effect on the total:

$${\Delta q}_{total}=q_{total}^{NG}-q_{total}^{HC}$$

was decomposed by stage, to attribute the NG-HC differences to early (S1) versus later (S2–S3) stages,

$${\Delta q}_{total}={\Delta q}_{S1}+{\Delta q}_{S2}+{\Delta q}_{S3}$$

where

$${\Delta q}_{s}=q_{s}^{NG}-q_{s}^{HC}$$

#### 3.9.3. Sensitivity to Initial Concentration (C_0_)

For experiments at C_0_ = 333.3 mg/L, removal was quantified using the same stage-wise percentage metric, %R_s_, computed for each stage. In addition, to compare the decorporation capacity across the entire regimen (NG or HC) as a function of initial cesium concentration, the regimen mean percentage removed ($\bar{{\%R}_{regimen}}$) was calculated for both C_0_ = 333.3 mg/L and C_0_ = 1000 mg/L and compared, following the equation:

$$\bar{{\%R}_{regimen}}=\frac{\sum_{s=1}^{3}\left(C_{0,s}-C_{s}\right)}{{3C}_{0}}$$

with the regimen being NG or HC.

#### 3.9.4. Single-Stage Controls (No Prior Exposure)

Single-stage tests at pH 6.8 and pH 7.2 (fresh tablet fragments), the same stage-wise definitions were used, yielding %R_single,6.8_ corresponding to the Cs percentage decorporated on pH 6.8 and %R_single,7.2_ corresponding to pH 7.2.

#### 3.9.5. Desorption in Mineralized Water

For the post-sequence desorption test, Cs released into the mineralized medium was quantified as C_des_ (mg/L) by ICP-OES. Desorption results were analyzed with the following parameters:

$$m_{des}=C_{des}\cdot V_{des}$$

$$\%desorbed=100\cdot \frac{m_{des}}{m_{captured}}$$

$$Desorbed\left(\frac{mg}{gPB}\right)=\frac{m_{des}}{m_{PB}}$$

with V_des_ = 0.015 L and m_PB_ = 0.075 g.

### 3.10. Statistical Analysis

All statistical analyses were conducted in Statgraphics Centurion XIX (v19) at a two-sided α = 0.05, unless otherwise stated. Data are presented as mean ±95% confidence interval (CI) unless indicated.

#### 3.10.1. Primary Endpoint

A two-way ANOVA with interaction (Formulation × Regimen) was fitted to qtotal. When the interaction was significant, simple effects analyses were performed as one-way ANOVAs within the relevant factor level. Tukey’s HSD was used for multiplicity-adjusted pairwise comparisons. Model assumptions were assessed via residual Q-Q plots (approximate normality) and Levene’s test (homogeneity of variances). No evidence of variance heterogeneity was observed, so the ANOVA models and Tukey’s HSD were retained. (Had heteroscedasticity been detected, results would have been corroborated using Welch’s ANOVA and/or non-parametric sensitivity analyses, together with the consistency of Tukey-type groupings).

#### 3.10.2. Stage-Wise Analysis

A balanced 4 × 2 factorial (n = 3 per cell) was considered: Formulation (Compression blend, PB tablet, PB-API bulk, Radiogardase^®^) × Regimen (NG: 1.2 → 6.8 → 7.2; HC: 4.0 → 6.8 → 7.2). For each stage separately, a two-way ANOVA was fitted. Least-squares means (95% CI) were obtained for Formulation × Regimen. Tukey’s HSD (α = 0.05) was used for multiplicity-adjusted pairwise comparisons where relevant.

To map where the total capacity is built, stage contribution fractions were computed and summarized by Formulation × Regimen (mean ± 95% CI).

Model assumptions were checked by residual Q-Q plots (approximate normality) and Levene’s test (homogeneity of variances). No evidence of variance heterogeneity was observed; therefore, ANOVA and Tukey’s HSD were retained.

#### 3.10.3. C_0_ Sensitivity Analysis

A general linear model (GLM) with fixed factors C_0_, Stage and Regimen and all two- and three-way interactions was fitted to %R_s_ based on Type III sums of squares. Least-square means with 95% CIs and interaction plots were obtained to aid interpretation.

In addition, one-way ANOVAs (factor C_0_ with 2 levels) were conducted separately for S1, S2, and S3 to test the effect of initial concentration within each stage.

Tukey’s HSD was used for multiplicity-adjusted pairwise comparisons; for the by-stage ANOVAs (two-level factor), the F-test on C_0_ provided the contrast. Model assumptions were checked via residual Q-Q plots (approximate normality) and Levene’s test (homogeneity of variances). No variance heterogeneity requiring robust alternatives was observed.

#### 3.10.4. Single-Stage Controls

Three one-way ANOVAs were performed: two of them, one at pH 6.8, comparing single vs. NG-S2 vs. HC-S2, and another at pH 7.2, comparing single vs. NG-S3 vs. HC-S3. These analyses were used to evaluate differences in Cs removal between ‘fresh’ samples and those previously exposed to a regimen sequence; and the final one comparing single 6.8 vs. single 7.2, to determine whether the typical PB pH-dependent behavior of fresh samples was confirmed, i.e., that the sample of pH 7.2 has a greater decorporating capacity than the pH 6.8 one [7,8,10,15]. Tukey’s HSD was used to adjusted pairwise tests. Assumptions were checked as above; Welch/Kruskal–Wallis was considered as sensitivity where appropriate.

#### 3.10.5. Desorption in Mineralized Water

For %desorbed and Desorbed (mg/g PB) (n = 3), one-variable analysis reported mean, SD and 95% CI. To formally substantiate a non-zero release, one-sample superiority tests (t-tests) were performed correspondently against 0% and 0 mg/g PB.

## 4. Conclusions

Taken together, these results show that the decorporation capacity of PB depends decisively on both the formulation and the pH history along GI transit. At an equal PB dose, the PB tablets manufactured at the University of Alcalá (and the equivalent compression blend) consistently outperform bulk PB-API, whereas Radiogardase^®^ performs clearly worse under normogastric conditions and only approaches the others under hypochlorhydria. This pattern does not reflect an average main effect of regimen but rather a Formulation × Regimen interaction: the regimen determines where the total is built. When first contact occurs at pH 1.2 (normogastric), capture in S1 is low and capacity is preserved for S2–S3, which are more favorable stages and where the tablet/blend formulations exhibit higher capacity, increasing the total. Conversely, at pH 4.0 (hypochlorhydria), S1 removes more but consumes capacity early, so S2–S3 contribute less; under NG, Radiogardase^®^ fails to compensate in later stages and its total falls.

The initial concentration (C_0_) sensitivity experiments and the single-stage controls reinforce this interpretation. Lowering C_0_ markedly increases the percentage removed in late stages, consistent with progressive saturation: the more PB captures up front, the less capacity remains for subsequent contacts. Indeed, with fresh PB at a single pH, 7.2 > 6.8 in percent removed, and single-stage tests exceed the corresponding stages in the sequential regimens, evidence that exposure history, not pH in isolation, limits S2–S3 effectiveness. The coherent exception is S2 in NG, where the preceding pH 1.2 stage contributes little saturation, and performance matches the fresh sample. This behavior aligns with the pH dependence reported in the literature, which places PB’s maximum exchange capacity for Cs^+^ around pH 7.5, with declining efficiency at more acidic pH values [7,8,10,15].

The desorption assay in mineralized water adds a direct practical implication: in the presence of Na^+^/K^+^/Ca^2+^/Mg^2+^ at levels representative of hard waters, substantial Cs release occurs from preloaded PB, whereas inter-stage washes with very soft water (Milli-Q) showed no detectable desorption. Accordingly, PB should be administered with very low mineralized water and not with hard/mineralized waters, both to maximize clinical efficacy and to minimize unwanted ionic exchange. Likewise, residues containing Cs-loaded PB should not be discharged unchecked into mineralized environments, as this could remobilize Cs; dedicated management consistent with applicable radiological/environmental regulations is required.

These conclusions arise from an in vitro model and should be validated in vivo. Even so, the stage-wise map and the agreement between primary and secondary outcomes support a clear message: formulation choice, the patient’s gastric acidity status, and the type of co-administered water are key determinants of PB’s real-world performance. Under normogastria, PB tablets (and the equivalent blend) offer the best expected performance relative to Radiogardase^®^; under hypochlorhydria, expected performances are more similar. Designing dosing and co-administration to preserve capacity for the most favorable stages and to avoid ionic competitors follows directly from the data.

## Figures and Tables

**Table 2:** Δq_s_ and Δq_total_ (NG-HC) by formulation (mg Cs/g PB).

Formulation	Δq_S1_ (mg Cs/g PB)	Δq_S2_ (mg Cs/g PB)	Δq_S3_ (mg Cs/g PB)	Δq_total_ (mg Cs/g PB)
Compression blend	−47.80 ± 5.03	+34.94 ± 11.82	+46.24 ± 9.43	+33.38 ± 13.05
PB tablets	−48.52 ± 5.03	+29.49 ± 11.82	+39.88 ± 9.43	+20.86 ± 13.05
PB-API (bulk)	−19.41 ± 5.03	+22.60 ± 11.82	+24.27 ± 9.43	+27.47 ± 13.05
Radiogardase^®^	−22.72 ± 5.03	−34.99 ± 11.82	−17.94 ± 9.43	−75.65 ± 13.05

**Table 3:** Contribution fractions fS1, fS2, fS3 (% of q_total_) by Formulation × Regimen (mean ± 95% CI).

Formulation	Regimen	f_S1_ (%)	f_S2_ (%)	f_S3_ (%)
Compression blend	NG	18.3% ± 0.6%	47.2% ± 1.3%	34.5% ± 0.9%
	HC	47.4% ± 4.2%	36.9% ± 5.5%	15.7% ± 9.7%
PB tablets	NG	17.0% ± 2.3%	48.8% ± 4.1%	34.2% ± 6.4%
	HC	44.0% ± 8.8%	38.8% ± 16.4%	17.3% ± 10.4%
PB-API (bulk)	NG	16.2% ± 2.6%	44.2% ± 8.7%	39.6% ± 6.1%
	HC	33.1% ± 2.6%	36.9% ± 9.2%	30.0% ± 9.1%
Radiogardase^®^	NG	9.3% ± 1.1%	45.3% ± 5.1%	45.4% ± 4.7%
	HC	18.5% ± 4.3%	45.7% ± 0.5%	35.8% ± 4.2%

**Table 4:** Composition and unit mass of the tested formulations.

Product Sample	PB (*w*/*w* %)	Glyceryl Behenate (*w*/*w* %)	HPMC (*w*/*w* %)	PVP K-30 (*w*/*w* %)	Microcrystalline Cellulose (*w*/*w* %)	Mean Unit Mass (mg)
PB-API	100.00	0.00	0.00	0.00	0.00	N/A
Pb tablets	71.43	1.00	1.00	3.00	23.57	700
Radiogardase^®^	100.00	0.00	0.00	0.00	0.00	500
Compression blend	71.43	1.00	1.00	3.00	23.57	N/A
Placebo (Excipient Blend)	0.00	3.50	3.50	10.50	82.50	N/A

**Table 5:** PB mass fraction and dose equalization parameters.

Product Sample	w_PB_ (*w*/*w*)	Target m_PB,test_ (mg)	m_product,test_ (mg) = m_PB,test_/w_PB_
PB-API	1.00	75.0	75.0
PB tablets	0.714	75.0	105.0
Radiogardase^®^ 500 mg (capsules)	1	75.0	75.0
Compression Blend	0.714	75.0	105.0
Placebo (Excipient Blend)	0.00	N/A	30.0

## Data Availability

The original contributions presented in this study are included in the article/Supplementary Materials. Further inquiries can be directed to the corresponding author.

## Supplementary Materials

The following supporting information can be downloaded at: https://www.mdpi.com/article/10.3390/ph18111702/s1, Table S1: Two-way ANOVA (Type III) for qt_otal_ (mg Cs/g PB) with factors Formulation and Regimen and partial η^2^; Table S2: Pairwise Tukey post-hoc comparisons of q_total_ among formulations within the normogastric (NG) regimen; Table S3: Pairwise Tukey post-hoc comparisons of q_total_ among formulations within the hypochlorhydria (HC) regimen; Table S4: Pairwise Tukey post-hoc comparisons of q_total_ (mg Cs/g PB) between formulations across regimens (NG-HC); Table S5: Two-way ANOVA (Type III) for q_S1_ (mg Cs/g PB) with partial η^2^ effect sizes; Table S6: Two-way ANOVA (Type III) for q_S2_ (mg Cs/g PB) with partial η^2^ effect sizes; Table S7: Two-way ANOVA (Type III) for q_S3_ (mg Cs/g PB) with partial η^2^ effect sizes; Table S8: (A) Table of Least Squares Means for q_S1_ (mg Cs/g PB). (B) Tukey HSD multiple comparisons for q_S1_ (mg Cs/g PB) - pairwise contrasts of formulations and regimens (95% confidence); Table S9: (A) Table of Least Squares Means for q_S2_ (mg Cs/g PB). (B) Tukey HSD multiple comparisons for q_S2_ (mg Cs/g PB) - pairwise contrasts of formulations and regimens (95% confidence); Table S10: (A) Table of Least Squares Means for q_S3_ (mg Cs/g PB). (B) Tukey HSD multiple comparisons for q_S3_ (mg Cs/g PB) - pairwise contrasts of formulations and regimens (95% confidence); Table S11: Three-way General Linear Model (Type III ANOVA) for %R_s_ (C_0_ sensitivity) showing main effects (C_0_, Stage, Regimen) and their interactions; Table S12: Tukey HSD multiple comparisons for %RS (C_0_ sensitivity) by stage (Factor C_0_); Table S13: (A) ANOVA for %R (single stage pH 6.8). (B) Table of means for %R (single stage pH 6.8) with 95.0% LSD intervals. (C) Tukey HSD multiple comparisons for %R (single stage pH 6.8) with 95% confidence; Table S14: (A) ANOVA for %R (single stage pH 7.2). (B) Table of means for %R (single stage pH 7.2) with 95.0% LSD intervals. (C) Tukey HSD multiple comparisons for %R (single stage pH 7.2) with 95% confidence; Table S15: (A) ANOVA for %R (single stage pH 6.8 vs pH 7.2). (B) Table of means for %R (single stage pH 6.8 vs pH 7.2) with 95.0% LSD intervals. (C) Tukey HSD multiple comparisons for %R (single stage pH 6.8 vs pH 7.2) with 95% confidence. Table S16: Descriptive statistics for %Cs desorbed under mineralized water condition; Table S17: One-sample non-inferiority tests versus zero for desorption in mineralized water).
