# Supplementary material for: Prussian Blue Tablets for Cesium Decorporation: pH-Dependent Performance Under Normogastric and Hypochlorhydric Conditions in an In Vitro Gastrointestinal Model
Source: Pharmaceuticals (Basel). 2025 Nov 10;18(11):1702. doi: 10.3390/ph18111702 (PMC12655675; doi:10.3390/ph18111702)
Supplement: Supplementary file 1 [file pharmaceuticals-18-01702-s001.zip › pharmaceuticals-3947137-supplementary.pdf]

pharmaceuticals

Supplementary material

Prussian Blue Tablets for Cesium Decorporation: pH-Dependent Performance Under Normogastric and Hypochlorhydric Conditions in an In Vitro Gastrointestinal Model

Borja Martínez-Alonso, Guillermo Torrado Durán, Hugo Pardo Laurel, Víctor Guarnizo-Herrero and M. Ángeles Peña Fernández *

Department of Biomedical Sciences, Faculty of Pharmacy, University of Alcalá (UAH), Campus Universitario, Crta. Madrid—Barcelona km. 33.600, 28771 Alcalá de Henares, Spain;
borja.martineza@uah.es (B.M.-A.); guillermo.torrado@uah.es (G.T.D.); hugo.pardo@edu.uah.es (H.P.L.); victor.guarnizo@uah.es (V.G.-H.)
* Correspondence: angeles.pena@uah.es; Tel.: +34-918854725; Fax: +34-918854658

Table S1. Two-way ANOVA (Type III) for q_{total} (mg Cs/g PB) with factors Formulation and Regimen and partial η^2.

Source	df	Sum of Squares	Mean Square	F-ratio	p-value	Partial η^2
Formulation	3	22525.4	7508.46	132.05	<0.0001	0.96
Regimen (NG vs HC)	1	13.79	13.79	0.24	0.6291	0.0015
Formulation × Regimen	3	12026	4008.94	70.50	<0.0001	0.90
Residual	16	909.787	56.8617			
Total	23	35475.8				

R^2 = 97.44 %, Adjusted R^2 = 96.31 %, Standard Error = 7.54 mg Cs/g PB.

Table S2. Pairwise Tukey post-hoc comparisons of q_{total} among formulations within the normogastric (NG) regimen.

Comparison	Difference (mg Cs/g PB)	95 % CI (±)	Significant (p < 0.05)
Compression blend – PB tablet	+5.07	±14.95	No
Compression blend – PB-API	+50.92	±14.95	Yes
Compression blend – Radiogardase®	+124.04	±14.95	Yes
PB tablet – PB-API	+45.85	±14.95	Yes
PB tablet – Radiogardase®	+118.97	±14.95	Yes
PB-API – Radiogardase®	+73.12	±14.95	Yes

Table S3. Pairwise Tukey post-hoc comparisons of q_{totall} among formulations within the hypochlorhydria (HC) regimen.

Comparison	Difference (mg Cs/g PB)	95 % CI (±)	Significant ($p < 0.05$)
Compression blend - PB tablet	-7.45	±23.52	No
Compression blend - PB-API	+45.01	±23.52	Yes
Compression blend - Radiogardase®	+15.01	±23.52	No
PB tablet - PB-API	+52.46	±23.52	Yes
PB tablet - Radiogardase®	+22.46	±23.52	No
PB-API - Radiogardase®	-30.00	±23.52	Yes

Table S4. Pairwise Tukey post-hoc comparisons of q_{total} (mg Cs/g PB) between formulations across regimens (NG - HC).

Formulation	NG - HC (mg Cs/g PB)	95 % CI (±)	Significant ($p < 0.05$)
Compression blend	+33.38	± 9.65	Yes
PB-API	+27.47	± 10.14	Yes
PB tablet	+20.86	± 12.97	Yes
Radiogardase®	-75.65	± 28.37	Yes

Table S5. Two-way ANOVA (Type III) for q_{S1} (mg Cs/g PB) with partial η^2 effect sizes.

Source	DF	Sum of Squares	Mean Square	F-ratio	p-value	Partial η^2
Regimen	1	7187.31	7187.31	852.36	<0.0001	0.98
Formulation	3	7707.3	2569.1	304.67	<0.0001	0.98
Regimen × Formulation	3	1109.89	369.965	43.84	<0.001	0.89
Residual	16	134.916	8.432			
Total (corrected)	23	16139.1				

R^2 = 99.16 %, adjusted R^2 = 98.80%, standard error = 2.90 mg Cs/g PB.

Table S6. Two-way ANOVA (Type III) for q_{S2} (mg Cs/g PB) with partial η^2 effect sizes.

Source	Sum of Squares	df	Mean Square	F-ratio	p-value	Partial η^2
Regimen	1015.80	1	1015.80	21.78	0.0003	0.58
Formulation	4091.85	3	1363.95	29.25	<0.0001	0.85
Regimen × Formulation	4723.18	3	1574.39	33.76	<0.0001	0.86
Residual	746.11	16	46.63			
Total (corrected)	10576.90	23				

R^2 = 92.95 %; adjusted R^2 = 89.86 %; standard error = 6.83 mg Cs/g PB.

Table S7. Two-way ANOVA (Type III) for q_{S3} (mg Cs/g PB) with partial η^2 effect sizes.

Source of variation	Sum of Squares	df	Mean Square	F-ratio	p-value	Partial η^2
Regimen	3205.09	1	3205.09	108.07	<0.0001	0.84
Formulation	19.03	3	6.34	0.21	0.8853	<0.01
Regimen × Formulation	3754.42	3	1251.47	42.20	<0.0001	0.83
Residual	474.54	16	29.66			
Total (corrected)	7453.07	23				

R^2 = 93.63 %; adjusted R^2 = 90.85 %; standard error = 5.45 mg Cs/g PB.

Table S8A. Table of Least Squares Means for q_{S1} (mg Cs/g PB).

Level	Count	Mean	Std. Error	Lower Limit	Upper Limit
GRAND MEAN	24	45.4319	0.592743	44.1753	46.6885
Regimen					
HC (pH 4.0→6.8→7.2)	12	62.7371	0.838266	60.9601	64.5142
NG (pH 1.2→6.8→7.2)	12	28.1267	0.838266	26.3496	29.9037
Formulation					
Compression blend	6	64.01	1.18549	61.4969	66.5231
PB tablet 500 mg	6	60.6017	1.18549	58.0685	63.1348
PB-API (bulk)	6	36.9193	1.18549	34.4061	39.4324
Radiogardase® 500 mg	6	20.1949	1.18549	17.6835	22.7098
Regimen by Formulation					
HC (pH 4.0) Compression blend	3	87.91	1.67653	84.3559	91.4641
HC (pH 4.0) PB tablet	3	84.86	1.67653	81.3059	88.4141
HC (pH 4.0) PB-API (bulk)	3	46.6219	1.67653	43.0678	50.1760
HC (pH 4.0) Radiogardase®	3	31.5567	1.67653	28.0026	35.1108
NG (pH 1.2) Compression blend	3	40.11	1.67653	36.5559	43.6641
NG (pH 1.2) PB tablet	3	36.3433	1.67653	32.7892	39.8974
NG (pH 1.2) PB-API (bulk)	3	27.2167	1.67653	23.6626	30.7708
NG (pH 1.2) Radiogardase®	3	8.83667	1.67653	5.28257	12.3908

Table S8B. Tukey HSD multiple comparisons for q_{S1} (mg Cs/g PB) - pairwise contrasts of formulations and regimens (95% confidence).

Contrast	Sig.	Difference	± Limits
Compression blend - PB tablet	No	3.40833	4.79879
Compression blend - PB-API (bulk)	Yes	27.0907	4.79879
Compression blend - Radiogardase®	Yes	43.8133	4.79879
PB tablet - PB-API (bulk)	Yes	23.6824	4.79879
PB tablet - Radiogardase®	Yes	40.405	4.79879
PB-API (bulk) - Radiogardase®	Yes	16.7226	4.79879
Regimen: NG (pH 1.2→6.8→7.2) - HC (pH 4.0→6.8→7.2)	Yes	-34.6105	2.51313

Table S9A. Table of Least Squares Means for q_{S2} (mg Cs/g PB).

Level	Count	Mean	Std. Error	Lower Limit	Upper Limit
GRAND MEAN	24	74.8351	1.39391	71.8801	77.79
Regimen					
HC (pH 4.0→6.8→7.2)	12	68.3293	1.97129	64.1503	72.5082
NG (pH 1.2→6.8→7.2)	12	81.3408	1.97129	77.1619	85.5198
Formulation					
Compression blend	6	85.9817	2.78783	80.1077	91.8961
PB tablet 500 mg	6	89.6133	2.78783	83.7383	95.5283
PB-API (bulk)	6	63.1533	2.78783	57.2783	69.0683
Radiogardase® 500 mg	6	60.5919	2.78783	54.682	66.5019
Regimen by Formulation					
HC (pH 4.0) Compression	3	68.51	3.94258	60.1527	76.8679
HC (pH 4.0) PB tablet	3	74.8667	3.94258	66.5087	83.2246
HC (pH 4.0) PB-API (bulk)	3	51.8532	3.94258	43.4953	60.2112
HC (pH 4.0) Radiogardase®	3	51.0867	3.94258	42.7287	59.4446
NG (pH 1.2) Compression	3	103.453	3.94258	95.0950	111.811
NG (pH 1.2) PB tablet	3	104.36	3.94258	96.0020	112.718
NG (pH 1.2) PB-API (bulk)	3	74.4533	3.94258	66.0964	82.8113
NG (pH 1.2) Radiogardase®	3	43.0967	3.94258	34.7387	51.4546

Table S9B. Tukey HSD multiple comparisons for q_{S2} (mg Cs/g PB) - pairwise contrasts of formulations and regimens (95% confidence).

Contrast	Sig.	Difference	± Limits
Compression blend - PB tablet	No	-3.63167	11.285
Compression blend - PB-API (bulk)	Yes	22.8284	11.285
Compression blend - Radiogardase®	Yes	25.3898	11.285
PB tablet - PB-API (bulk)	Yes	26.46	11.285
PB tablet- Radiogardase®	Yes	29.0214	11.285
PB-API (bulk) - Radiogardase®	No	2.56137	11.285
Regimen: NG (pH 1.2→6.8→7.2) - HC (pH 4.0→6.8→7.2)	Yes	13.0116	5.90994

Table S10A. Table of Least Squares Means for q_{S3} (mg Cs/g PB).

Level	Count	Mean	Std. Error	Lower Limit	Upper Limit
GRAND MEAN	24	53.0447	1.11165	50.6881	55.4013
Regimen					
HC (pH 4.0→6.8→7.2)	12	41.4885	1.57211	38.1557	44.8212
NG (pH 1.2→6.8→7.2)	12	64.6008	1.57211	61.2681	67.9336
Formulation					
Compression blend	6	52.3917	2.2233	47.6785	57.1049
PB tablet 500 mg	6	53.3617	2.2233	48.6485	58.0749
PB-API (bulk)	6	54.3501	2.2233	49.6369	59.0633
Radiogardase® 500 mg	6	52.0752	2.2233	47.3620	56.7884
Regimen by Formulation					
HC (pH 4.0) Compression blend	3	29.2733	3.14422	22.6079	35.9388
HC (pH 4.0) PB tablet	3	32.2067	3.14422	25.5412	38.8721
HC (pH 4.0) PB-API (bulk)	3	42.4125	3.14422	35.7468	48.8799
HC (pH 4.0) Radiogardase®	3	61.0417	3.14422	54.3763	67.7071
NG (pH 1.2) Compression blend	3	75.51	3.14422	68.8446	82.1755
NG (pH 1.2) PB tablet	3	74.3033	3.14422	67.6379	80.9687
NG (pH 1.2) PB-API (bulk)	3	66.4867	3.14422	59.8212	73.1521
NG (pH 1.2) Radiogardase®	3	43.1033	3.14422	36.4379	49.7688

Table S10B. Tukey HSD multiple comparisons for q_{S3} (mg Cs/g PB) - pairwise contrasts of formulations and regimens (95% confidence).

Contrast	Sig.	Difference	± Limits
Compression blend - PB tablet	No	-0.97	8.99981
Compression blend - PB-API (bulk)	Yes	-1.95842	8.99981
Compression blend - Radiogardase®	Yes	0.316467	8.99981
PB tablet - PB-API (bulk)	Yes	-0.988425	8.99981
PB tablet- Radiogardase®	Yes	1.28647	8.99981
PB-API (bulk) - Radiogardase®	No	2.27489	8.99981
Regimen: NG (pH 1.2→6.8→7.2) - HC (pH 4.0→6.8→7.2)	Yes	23.1124	4.7132

Table S11. Three-way General Linear Model (Type III ANOVA) for %R_s (C_0 sensitivity) showing main effects (C_0, Stage, Regimen) and their interactions.

Source	df	Sum of Squares	Mean Square	F-ratio	p-value	Partial η^2
C_0 (mg L^{-1})	1	8163.46	8163.46	600.95	<0.0001	0.96
Stage	2	3091.77	1545.88	113.80	<0.0001	0.90
Regimen	1	25.1823	25.18	1.85	0.186	0.07
C_0 × Stage	2	399.809	199.90	14.72	0.0001	0.55
C_0 × Regimen	1	29.2876	29.29	2.16	0.155	0.08
Stage × Regimen	2	6030.49	3015.24	221.97	<0.0001	0.95
C_0 × Stage × Regimen	2	371.948	185.99	13.69	0.0001	0.53
Residual	24	326.022	13.58			
Total (Corrected)	35	18438				

R^2 = 98.23 %; adjusted R^2 = 97.42 %; standard error = 3.69 mg Cs/g PB.

Table S12. Tukey HSD multiple comparisons for %RS (C_0 sensitivity) by stage (Factor C_0).

**S1**			
**C_0 (mg/L)**	**Count**	**Mean**	
1000	6	30.3008	
333.3	6	51.0177	
**Contrast**	**Sig.**	**Difference**	**Limits**
333.3 - 1000	No	20.7169	25.2108
**S2**			
**C_0 (mg/L)**	**Count**	**Mean**	
1000	6	4480.67	
333.3	6	79.051	
**Contrast**	**Sig.**	**Difference**	**Limits**
333.3 - 1000	Yes	34.2184	11.2655
**S3**			
**C_0 (mg/L)**	**Count**	**Mean**	
1000	6	26.6808	
333.3	6	62.0974	
**Contrast**	**Sig.**	**Difference**	**Limits**
333.3 - 1000	Yes	35.4166	18.9737

Table S13A. ANOVA for %R (single stage pH 6.8).

Source	df	Sum of Squares	Mean Square	F-ratio	p-value
Between groups	2	534.616	267.308	16.65	0.0036
Within groups	6	96.3537	16.059		
Total (Corr.)	8	630.97			

Table S13B. Table of means for %R (single stage pH 6.8) with 95.0% LSD intervals.

Group	Count	Mean	Stnd. Error (pooled s)	Lower limit	Upper limit
HC_seq	3	37.4333	2.31365	33.4302	41.4365
Stage × Regimen	3	52.18	2.31365	48.1768	56.1832
Single	3	55.015	2.31365	51.0118	59.0182
Total	9	48.2094			

Table S13C. Tukey HSD multiple comparisons for %R (single stage pH 6.8) with 95% confidence.

Contrast	Sig.	Difference	± Limits
HC_seq - NG_seq	Yes	14.7467	10.0395
HC_seq - Single	Yes	-17.5817	10.0395
NG_seq - Single	No	-2.835	10.0395

Table S14A. ANOVA for %R (single stage pH 7.2).

Source	df	Sum of Squares	Mean Square	F-ratio	p-value
Between groups	2	4091.4	2045.7	184.55	<0.0001
Within groups	6	66.5087	11.0848		
Total (Corr.)	8	4157.91			

Table S14B. Table of means for %R (single stage pH 7.2) with 95.0% LSD intervals.

Group	Count	Mean	Stnd. Error (pooled s)	Lower limit	Upper limit
HC_seq	3	16.71	1.92222	13.3841	20.0359
Stage × Regimen	3	36.6517	1.92222	33.3258	39.9776
Single	3	68.4833	1.92222	65.1574	71.8092
Total	9	40.615			

Table S14C. Tukey HSD multiple comparisons for %R (single stage pH 7.2) with 95% confidence.

Contrast	Sig.	Difference	± Limits
HC_seq - NG_seq	Yes	-19.9417	8.34095
HC_seq - Single	Yes	-51.7733	8.34095
NG_seq - Single	Yes	-31.8317	8.34095

Table S15A. ANOVA for %R (single stage pH 6.8 vs pH 7.2).

Source	df	Sum of Squares	Mean Square	F-ratio	p-value
Between groups	1	272.094	272.094	145.22	0.0003
Within groups	4	7.49472	1.87368		
Total (Corr.)	5	279.589			

Table S15B. Table of means for %R (single stage pH 6.8 vs pH 7.2) with 95.0% LSD intervals.

Group	Count	Mean	Stnd. Error (pooled s)	Lower limit	Upper limit
6.8	3	55.015	0.790291	53.4635	56.5665
7.2	3	68.4833	0.790291	66.9318	70.0349
Total	6	61.7492			

Table S15C. Tukey HSD multiple comparisons for %R (single stage pH 6.8 vs pH 7.2) with 95% confidence.

Contrast	Sig.	Difference	± Limits
6.8 - 7.2	Yes	-13.4683	3.10308

Table S16. Descriptive statistics for %Cs desorbed under mineralized water condition.

Statistic	Value	95% CI (Lower - Upper)
Mean (%)	41.37	40.27 - 42.48
Standard deviation (%)	0.44	0.23 - 2.79
Minimum (%)	40.97	
Maximum (%)	41.85	

Table S17. One-sample non-inferiority tests versus zero for desorption in mineralized water).

Endpoint	Mean ± SD	Lower 95% CI	Test statistic (t)	p-value	Conclusion
%Cs desorbed	41.37 ± 0.44	40.62	161.195	<0.0001	Non-inferiority vs 0%
Cs desorbed (mg/g PB)	88.55 ± 3.52	82.62	43.5339	0.0003	Non-inferiority vs 0 mg/g
